# Bimodal antagonism of PKA signalling by ARHGAP36

**DOI:** 10.1038/ncomms12963

**Published:** 2016-10-07

**Authors:** Rebecca L. Eccles, Maciej T. Czajkowski, Carolin Barth, Paul Markus Müller, Erik McShane, Stephan Grunwald, Patrick Beaudette, Nora Mecklenburg, Rudolf Volkmer, Kerstin Zühlke, Gunnar Dittmar, Matthias Selbach, Annette Hammes, Oliver Daumke, Enno Klussmann, Sylvie Urbé, Oliver Rocks

**Affiliations:** 1 Max-Delbrück-Center for Molecular Medicine, Robert-Roessle-Straße 10, 13125 Berlin, Germany; 2 Berlin Institute of Health (BIH), Kapelle-Ufer 2, 10117 Berlin, Germany; 3 Leibniz-Institut für Molekulare Pharmakologie, Robert-Roessle-Straße 10, 13125 Berlin, Germany; 4 Institute for Chemistry and Biochemistry, Freie Universität Berlin, Takustraße 6, 14195 Berlin, Germany; 5 DZHK, German Centre for Cardiovascular Research, Oudenarder Straße 16, 13347 Berlin, Germany; 6 Cellular and Molecular Physiology, Institute of Translational Medicine, University of Liverpool, Liverpool L69 3BX, UK

## Abstract

Protein kinase A is a key mediator of cAMP signalling downstream of G-protein-coupled receptors, a signalling pathway conserved in all eukaryotes. cAMP binding to the regulatory subunits (PKAR) relieves their inhibition of the catalytic subunits (PKAC). Here we report that ARHGAP36 combines two distinct inhibitory mechanisms to antagonise PKA signalling. First, it blocks PKAC activity via a pseudosubstrate motif, akin to the mechanism employed by the protein kinase inhibitor proteins. Second, it targets PKAC for rapid ubiquitin-mediated lysosomal degradation, a pathway usually reserved for transmembrane receptors. ARHGAP36 thus dampens the sensitivity of cells to cAMP. We show that PKA inhibition by ARHGAP36 promotes derepression of the Hedgehog signalling pathway, thereby providing a simple rationale for the upregulation of ARHGAP36 in medulloblastoma. Our work reveals a new layer of PKA regulation that may play an important role in development and disease.

## Abstract (web summary)

Protein kinase A (PKA) is a key mediator of cyclic AMP signalling. Here, Eccles *et al*. show that ARHGAP36 antagonizes PKA by acting as a kinase inhibitor and targeting the catalytic subunit for endolysosomal degradation, thus reducing sensitivity of cells to cAMP and promoting Hedgehog signalling.

Protein kinase A (PKA) is a widely expressed serine/threonine kinase regulating essential cellular processes important in development, metabolism, memory formation and proliferation^1^. The inactive tetrameric holoenzyme comprises a dimer of regulatory subunits (PKAR), each of which binds a catalytic subunit (PKAC) in its active-site cleft, thereby blocking kinase activity and access to substrates. PKA activation occurs on binding of cyclic AMP (cAMP) to PKAR, causing the release of PKAC and thus allowing phosphorylation of numerous downstream proteins. Misregulated PKA signalling is linked to various human diseases and recent studies have identified pathway-activating mutations in both PKAR and PKAC as well as in other upstream components in a number of cancers^2^,^3^,^4^,^5^,^6^,^7^,^8^. Since PKA regulates a myriad of cellular responses, precise spatiotemporal control is required to ensure its signalling specificity. A-kinase-anchoring proteins (AKAPs) bind PKAR and target the holoenzyme to distinct subcellular compartments. AKAPs serve as scaffolds to place PKA in the vicinity of specific substrates together with phosphodiesterases, phosphatases or components of wider signalling networks, thereby providing tailored cAMP signalling units^9^,^10^. In addition, PKA activity can be negatively regulated by a family of small, heat-stable protein kinase inhibitor (PKI) proteins, each containing a pseudosubstrate motif by which they bind PKAC with high affinity^11^. On the other hand, cAMP-dependent proteasomal degradation of PKAR can positively modulate PKA activity by increasing the pool of free PKAC. This mechanism facilitates long-term memory formation^12^ and involves the E3 ubiquitin ligase Praja2 (ref. 13). Ubiquitin-mediated degradation of PKAC has so far not been described, although regulated proteolysis is a common mechanism for downregulating activated protein kinases^14^,^15^.

Here we report the identification of the Rho GTPase-activating protein (RhoGAP) family member ARHGAP36 as a potent antagonist of PKA signalling. ARHGAP36 is not only a PKA pseudosubstrate inhibitor but also targets PKAC for ubiquitin-dependent proteolysis. Unexpectedly for a cytosolic protein, PKAC degradation is not mediated by the proteasome but by the endolysosomal system. This pathway typically mediates degradation of activated receptor tyrosine kinases^16^,^17^. ARHGAP36 has previously been shown to activate the Hedgehog (Hh) signalling pathway and is upregulated in a subset of medulloblastoma, suggesting an important role in tumourigenesis^18^. PKA is a master negative regulator of the Hh pathway^19^,^20^, therefore PKA inhibition by ARHGAP36 provides a straightforward mechanism for this observation. Our study thus defines a new paradigm for negative PKA regulation with implications in health and disease.

## Results

### ARHGAP36 interacts with PKAC

**Figure 1:**
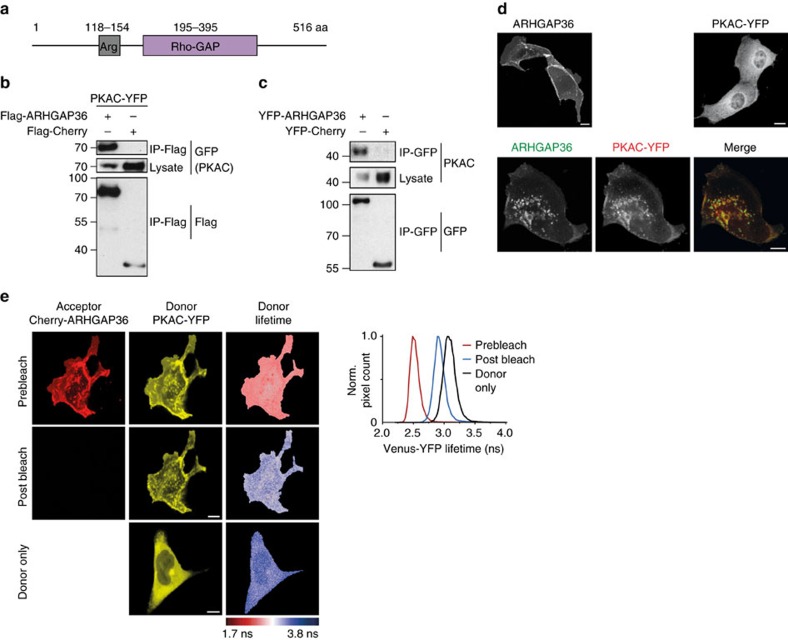
ARHGAP36 interacts with PKAC. (**a**) Schematic representation of human ARHGAP36 isoform 2 (UniProt ID: Q6ZRI8-2). (**b**) HEK293T cells were transfected with PKAC-YFP and Flag-ARHGAP36 or Flag-Cherry control. Lysates were immunoprecipitated using a Flag antibody and immunoblotted with GFP or Flag antibodies. Note the reduction of PKAC in presence of ARHGAP36. (**c**) HEK293T cells were transfected with YFP-ARHGAP36 or YFP-Cherry control. Lysates were immunoprecipitated using a GFP antibody and immunoblotted with GFP or PKAC antibodies. As in **b**, note the reduction of PKAC in the presence of ARHGAP36. (**d**) Confocal live micrographs of MDCK cells expressing CFP-ARHGAP36 and PKAC-YFP, either alone or together. Scale bars, 10μm. (**e**) FLIM–FRET measurements in MDCK cells expressing PKAC-YFP (donor) either alone, or together with mCherry-ARHGAP36 (acceptor) before and after acceptor photobleaching. Shown are YFP and mCherry confocal images and pseudocoloured donor fluorescence lifetime maps. Scale bars, 10μm. Right panel: corresponding histograms of the prebleach (red) and postbleach (blue) Venus-YFP lifetime values together with the donor only control sample (black).

In a systematic mass spectrometric (MS) analysis of the Rho GTPase regulatory proteins, we identified ARHGAP36 as a new binding partner of PKAC (O.R., manuscript in preparation). ARHGAP36 has five annotated isoforms with molecular masses between 46 and 61 kDa, which vary only in their extreme N-terminal portion. All contain an arginine-rich region followed by a RhoGAP domain (Fig. 1a; Supplementary Fig. 1).

To further investigate this interaction, we first confirmed the association of overexpressed ARHGAP36 both with ectopic and endogenous PKAC by co-immunoprecipitation (Fig. 1b,c). In Madin–Darby canine kidney II (MDCK) cells, ARHGAP36 expressed alone was concentrated at the plasma membrane, while PKAC appeared largely cytosolic. However, upon coexpression, PKAC was completely recruited by ARHGAP36, with both proteins exhibiting a pronounced co-localization on vesicles (Fig. 1d). We further analysed the interaction by fluorescence lifetime imaging microscopy-based fluorescence resonance energy transfer (FLIM–FRET)^21^. A PKAC-YFP donor and mCherry-ARHGAP36 acceptor fluorophore exhibited substantial FRET as apparent from the decreased donor fluorescence lifetime (Fig. 1e). Acceptor photobleaching restored the long-lived donor lifetime, confirming the direct interaction of ARHGAP36 and PKAC.

### ARHGAP36 binds PKAC via a pseudosubstrate motif

**Figure 2:**
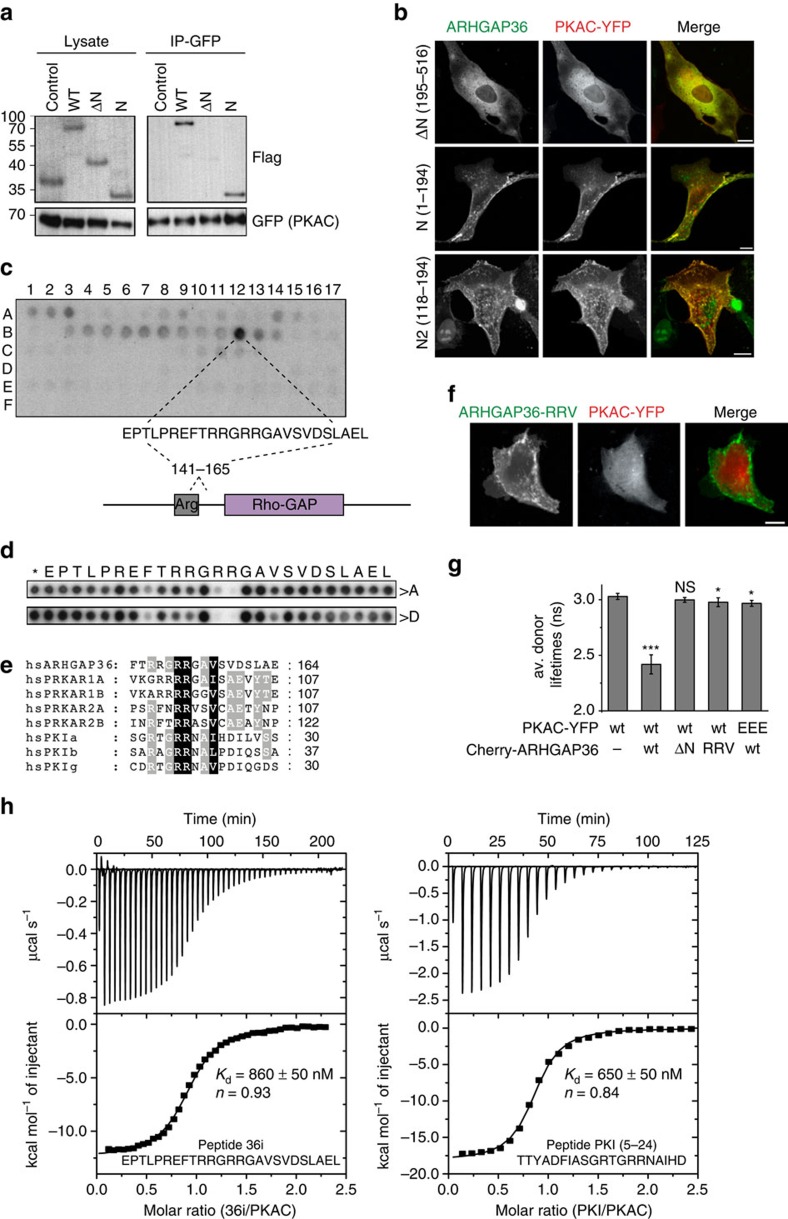
ARHGAP36 interacts with PKAC via a pseudosubstrate domain. (**a**) HEK293T cells were transfected with PKAC-YFP together with Flag-tagged wild-type ARHGAP36 (WT), 195–516 (ΔN), 1–194 (N) or Flag-Cherry control. Lysates were immunoprecipitated using a GFP antibody and immunoblotted using GFP or Flag antibodies. (**b**) Confocal live micrographs of MDCK cells expressing PKAC-YFP together with CFP-ΔN, CFP-N or CFP-N2 (118–194). Nuclear enrichment of ARHGAP36-N2, bottom left, may be facilitated by the arginine-rich sequence. Scale bars, 10 μm. (**c**) Immobilized peptide ‘spots', overlapping 25-mer peptides each shifted along by 5 aa in the entire ARHGAP36 sequence, were probed for interaction with GST-PKAC and immunoblotted using a GST antibody. The sequence of the spot with strongest interaction is shown. (**d**) Alanine and aspartate scans of the spot indicated in **c** were treated the same as in **c**. Asterisk indicates wild-type sequence spot. (**e**) Alignment of human ARHGAP36 with the human isoforms of PRKAR and PKI revealing its pseudosubstrate motif RRxAY. (**f**) Confocal live micrographs of MDCK cells coexpressing CFP-ARHGAP36-RRV and PKAC-YFP. Scale bars, 10 μm. (**g**) Average Venus-YFP fluorescence lifetimes of MDCK cells expressing PKAC-YFP alone or together with mCherry-ARHGAP36, -ΔN or -RRV, or PKAC-EEE-YFP (E127A/E170A/E230A) together with mCherry-ARHGAP36 (*n*≥4 each). Error bars denote mean±s.d. ***P<0.001, *P<0.05; NS, not significant; Student's *T*-test versus PKAC-YFP donor alone. (**h**) ITC measurements were performed in the presence of 2 mM ATP analogue adenylyl-imidodiphosphate (AMPPNP). A concentration of 30 μM His-PKAC solution (cell) was titrated with 507 μM PKI or 351 μM 36i (syringe) until saturation was reached using 8 or 6 μl injections, respectively (the first injection was always carried out with half the volume and omitted from data analysis). 36i and PKI bound to PKAC●AMPPNP with almost identical affinities. The measured values are in a similar range as previously reported for the PKAC–PKI interaction in the presence of other ATP analogues or ADP^71^.

We mapped the interaction site with PKAC to the N-terminus of ARHGAP36. Its deletion rendered ARHGAP36 cytosolic and abolished the interaction with PKAC (Fig. 2a,b,g; Supplementary Fig. 2a). The N terminus and, furthermore, just 77 aa (118–194 ‘N2') containing the arginine-rich region were sufficient both for interaction and co-localization of ARHGAP36 and PKAC. Peptide spot experiments pinpointed the interaction site to a 25 aa sequence (Fig. 2c). Subsequent alanine and aspartate scanning of this sequence revealed the requirement of two arginines R153/R154 for PKAC binding (Fig. 2d). We noticed that the sequence adjacent to the arginines (RRGAV) matches the consensus motif (R-R-*X*-S/(A/G)-*Y*) for PKAC substrates/inhibitors, where X is variable, Y a hydrophobic residue and S/(A/G) the phosphorylation/pseudophosphorylation site, respectively^1^,^22^ (Fig. 2e). This motif, shared by all PKAR subunits and PKIs, docks into the acidic active-site cleft of PKAC^1^,^23^. Aspartate mutation of three residues R153D/R154D/V157D (RRV) within this site impaired the co-localization and interaction of ARHGAP36 with PKAC (Fig. 2f,g; Supplementary Fig. 2b,c). Likewise, substitution of three corresponding glutamate residues E127A/E170A/E230A (EEE) in the acidic active-site cleft of PKAC, shown to ion pair with the pseudosubstrate motif arginines of PKAR^24^, compromised the interaction of PKAC with ARHGAP36 (Fig. 2g; Supplementary Fig. 2d–f). Using isothermal titration calorimetry (ITC), we found that a synthesised peptide comprising the 25 aa ARHGAP36 pseudosubstrate motif (36i, Fig. 2c) bound with high nanomolar affinity to purified PKAC in the presence of the non-hydrolysable ATP analogue adenylyl-imidodiphosphate (Fig. 2h; Supplementary Fig. 2g,h). A similar value was obtained with a PKI-derived peptide (amino acids 5-24), supporting our notion of related binding modes for these two peptides. These experiments demonstrate the direct interaction between ARHGAP36 and PKAC, mediated by a pseudosubstrate motif on ARHGAP36.

### ARHGAP36 inhibits PKAC catalytic activity

**Figure 3:**
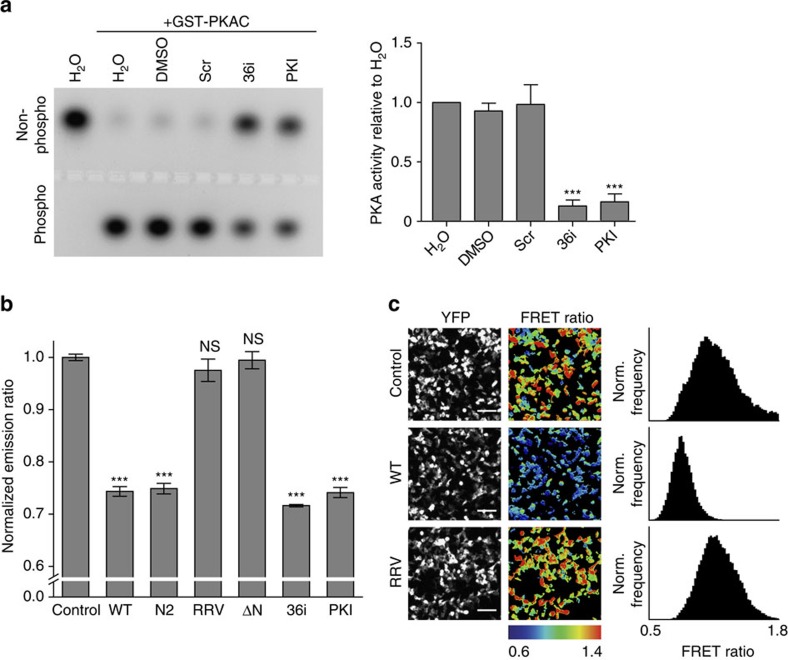
ARHGAP36 inhibits PKAC. (**a**) Recombinant PKAC (25 ng) was incubated with 10 μM scrambled, 36i or PKI peptide, and its activity determined by its ability to phosphorylate the substrate peptide PepTag A1. Phosphorylated and non-phosphorylated peptide were separated by agarose electrophoresis, and densitometrically analysed. Representative gel shown. Recombinant PKAC activity is shown as the ratio of phosphorylated to non-phosphorylated peptide (mean of eight repeats±s.e.m., ***P<0.001, Student's *T*-test versus all three controls). (**b**) AKAR4-NES FRET sensor was expressed together with Cherry control, Cherry-ARHGAP36 (WT), the indicated mutants or 36i-Cherry or PKI-Cherry in HEK293T cells. Cells were serum starved for 5 h, treated with 10 μM Forskolin and 100 μM IBMX for 30 min, and subsequently imaged. Mean FRET emission ratio (acceptor intensity/donor intensity) of three independent experiments normalized to control±s.d. ***P<0.001, NS, not significant, versus control and RRV. (**c**) Representative images of HEK293T cells expressing AKAR4-NES FRET sensor together with Cherry control, Cherry-ARHGAP36 (WT) or Cherry-ARHGAP36-RRV. Cells were treated as in **b**. Shown are YFP intensity images and pseudocoloured FRET ratio images (acceptor intensity/donor intensity) reflecting the relative PKA activity levels. Scale bars, 100 μm. The histograms show the pixel distribution within the FRET emission ratio images.

As ARHGAP36 and PKAR share the same binding site on PKAC, we next investigated whether ARHGAP36 also inhibits the catalytic activity of PKAC, similar to the mode of PKAC inhibition by PKI^25^. First, we performed an *in vitro* kinase assay with recombinant PKAC. Addition of 36i peptide strongly reduced the fraction of phosphorylated PKA substrate (Fig. 3a). A similar extent of kinase inhibition was achieved with equal concentrations of PKI (5–24) peptide. We further analysed the impact of ARHGAP36 on PKA activity in cells using a genetically encoded FRET-based PKA biosensor (AKAR4-NES)^26^. Coexpression of full-length ARHGAP36 with AKAR4-NES caused a strong decrease in PKA activity in stimulated cells, as evident from the reduction in mean FRET emission ratio (25.7±0.9%, ±indicates s.d., *n*=3, Fig. 3b,c). ARHGAP36-ΔN or ARHGAP36-RRV did not alter PKA activity levels, whereas ARHGAP36-N2, containing the pseudosubstrate site, decreased PKA activity to the same extent as the full-length form (25.1±1.0%, ±indicates s.d., *n*=3). Constructs encoding 36i, or full-length PKI, also significantly reduced PKA activity (by 28.4±0.2% and 25.9±1.0%, respectively, ±indicates s.d., *n*=3). The above experiments collectively demonstrate that ARHGAP36 is a pseudosubstrate inhibitor of PKAC with potency comparable to PKI.

### ARHGAP36 causes depletion of PKAC

**Figure 4:**
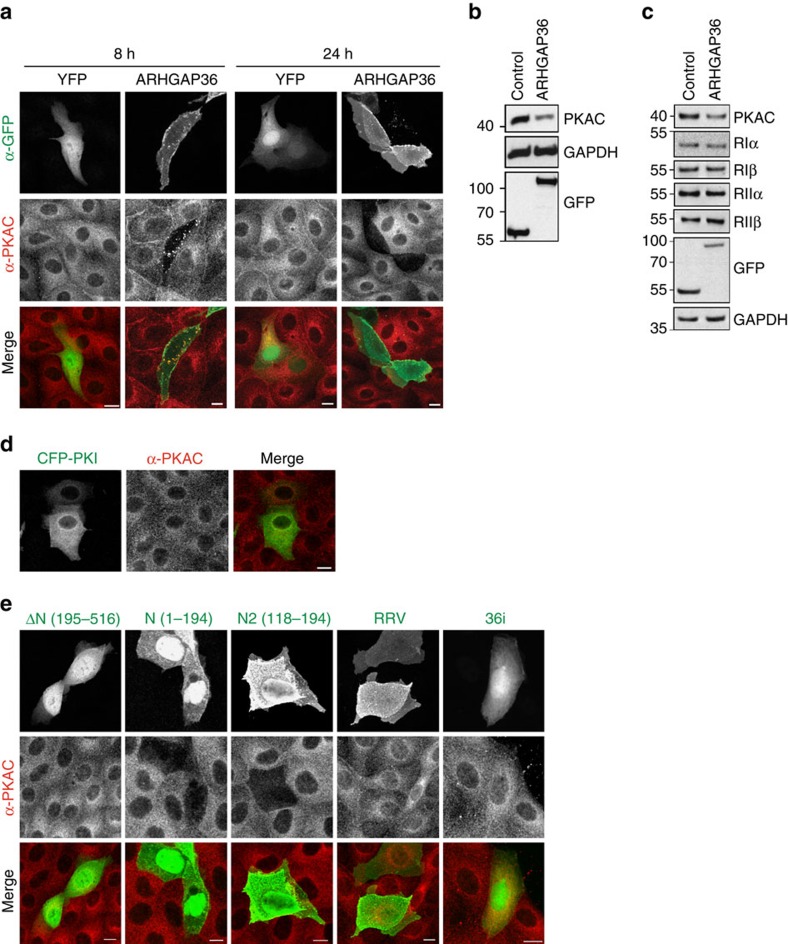
ARHGAP36 downregulates PKAC levels. (**a**) MDCK cells transfected with YFP-ARHGAP36, or a YFP control, were fixed after 8 or 24 h and subjected to immunofluorescence using antibodies against GFP and PKAC. Images were collected by confocal microscopy. Scale bars, 10 μm. (**b**) HEK293T cells were transfected with YFP-ARHGAP36 or YFP-Cherry control for 24 h. Lysates were immunoblotted with the indicated antibodies. (**c**) HEK293T cells were transfected with YFP-ARHGAP36 or YFP control. Lysates were immunoblotted with antibodies against RIα, RIβ RIIα, RIIβ, GAPDH and GFP. (**d**) MDCK cells transfected with CFP-PKI were fixed after 24 h and subjected to immunofluorescence as in **a**. Scale bar, 10 μm. (**e**) MDCK cells transfected with YFP-ΔN (195–516), YFP-N (1–194), YFP-N2 (118–195), YFP-RRV or Cherry-36i were fixed after 24 h and subjected to immunofluorescence as in **a**. 36i signal was not amplified using antibodies. Scale bars, 10 μm.

We consistently noticed a marked downregulation of PKAC upon ARHGAP36 expression (Fig. 1b,c) and therefore wondered whether ARHGAP36 also controlled PKAC stability. Protein levels of endogenous PKAC in YFP-ARHGAP36-expressing MDCK cells were already markedly reduced just 8 h after transfection, with the two proteins co-localizing on vesicles (Fig. 4a). After 24 h of ARHGAP36 transfection, PKAC was almost completely depleted. Downregulation of endogenous PKAC could also be seen in HEK293T cells overexpressing ARHGAP36 (Fig. 4b). PKAC depletion is not secondary to an effect on PKAR: ARHGAP36 overexpression did not affect protein levels of PKAR isoforms in either HEK293T or MDCK cells (Fig. 4c; Supplementary Fig. 3a). We then asked whether the simple displacement of PKAC from PKAR, by competitive binding of a pseudosubstrate, is sufficient to promote its downregulation. However, expression of full-length PKI did not affect endogenous PKAC protein levels, suggesting the ability to downregulate PKAC is a specific feature of ARHGAP36 (Fig. 4d; Supplementary Fig. 3b). Direct interaction with ARHGAP36 is required for PKAC downregulation as expression of ARHGAP36-ΔN or ARHGAP36-RRV failed to deplete endogenous PKAC. 36i, encoding only the pseudosubstrate motif, was equally without effect, while ARHGAP36-N and ARHGAP36-N2 promoted efficient depletion of PKAC (Fig. 4e; Supplementary Fig. 3b). Pseudosubstrate binding is thus necessary but not sufficient to promote a decrease in PKAC levels. The RRGAV-binding motif on ARHGAP36 mediates the interaction with PKAC, while the downregulatory properties are encoded nearby.

### ARHGAP36 targets PKAC for lysosomal degradation

**Figure 5:**
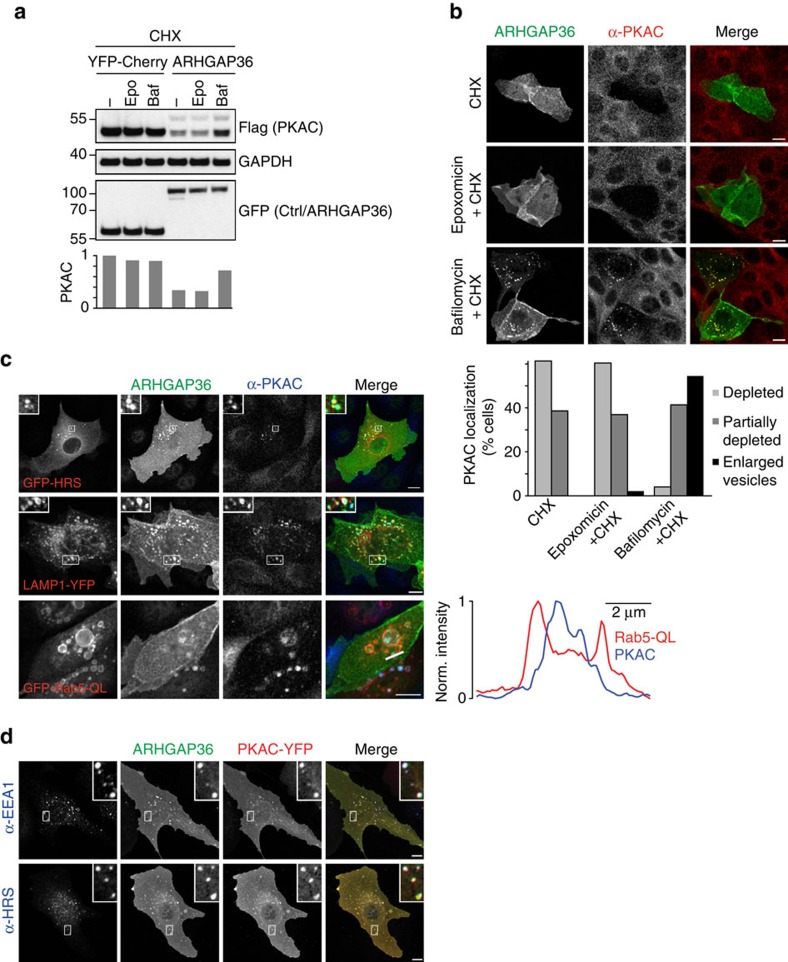
ARHGAP36 targets PKAC for lysosomal degradation. (**a**) HEK293T cells were transfected with PKAC-Flag and YFP-ARHGAP36 or YFP-Cherry control as indicated. Before collecting, cells were pretreated for 30 min with epoxomicin (50 nM) or bafilomycin (100 nM), before cycloheximide (CHX; 50 μg ml^−1^) addition for a further 6 h. Lysates were immunoblotted with the indicated antibodies. Flag-PKAC levels were densitometrically evaluated and normalized to the first lane shown. (**b**) MDCK cells were transfected with YFP-ARHGAP36 or YFP control for 8 h and, where indicated, were pretreated for 30 min with epoxomycin (100 nM) or bafilomycin (100 nM), before CHX (1 μg ml^−1^) addition for a further 8 h. Cells were then fixed after a total of 16 h transfection and subjected to immunofluorescence using antibodies against GFP and PKAC. Images were collected by confocal microscopy. Scale bars, 10 μm. Graph shows the percentage of YFP-ARHGAP36-transfected cells with the respective phenotype, 100 cells counted per condition. (**c**) Confocal micrographs of MDCK cells transfected with Flag-ARHGAP36 together with GFP-HRS, LAMP1-YFP or GFP-Rab5-QL. Cells were fixed and subjected to immunofluorescence using antibodies against GFP, Flag and PKAC. Scale bars, 10 μm. For Rab5-QL, line scan fluorescence intensity profiles are shown on the right. In red: Rab5-QL, in blue: PKAC. (**d**) Confocal micrographs of U2OS cells transfected with PKAC-YFP and Flag-ARHGAP36. Cells were fixed and subjected to immunofluorescence using antibodies against GFP, Flag, EEA1 and HRS. Scale bars, 10 μm.

We wondered which of the main cellular protein degradation pathways, the proteasomal or the endolysosomal system, were responsible for PKAC downregulation in ARHGAP36-transfected cells. We therefore treated cells with the protein synthesis inhibitor cycloheximide and analysed PKAC protein levels in the presence of inhibitors against these pathways. Surprisingly, we saw no effect with the proteasome inhibitor epoxomicin (Fig. 5a; Supplementary Fig. 4). Instead, PKAC levels were partially rescued by treatment with bafilomycin, which blocks the acidification of the endolysosomal compartment^27^. Similarly, in MDCK cells, cycloheximide treatment caused a loss of endogenous PKAC that could not be rescued by epoxomicin (Fig. 5b). In contrast, addition of bafilomycin resulted in a build-up of PKAC together with ARHGAP36 on enlarged vesicles. In the presence of YFP-ARHGAP36, endogenous PKAC co-localized with the coexpressed early endosomal/multivesicular body (MVB) marker HRS and the late endosomal/lysosomal marker LAMP1 (Fig. 5c). Coexpressed ARHGAP36 and PKAC also co-localized with the endogenous early endosome marker EEA1 and HRS (Fig. 5d). Moreover, when we coexpressed ARHGAP36 with constitutively active Rab5 Q79L to induce enlarged endosomes and thus to facilitate resolution of the MVB lumen from the surrounding limiting membrane^28^, we clearly observed endogenous PKAC inside these vesicles (Fig. 5c). These experiments show that ARHGAP36 targets PKAC, a cytosolic protein, for lysosomal degradation.

### PKAC degradation requires ubiquitylation and the ESCRT pathway

**Figure 6:**
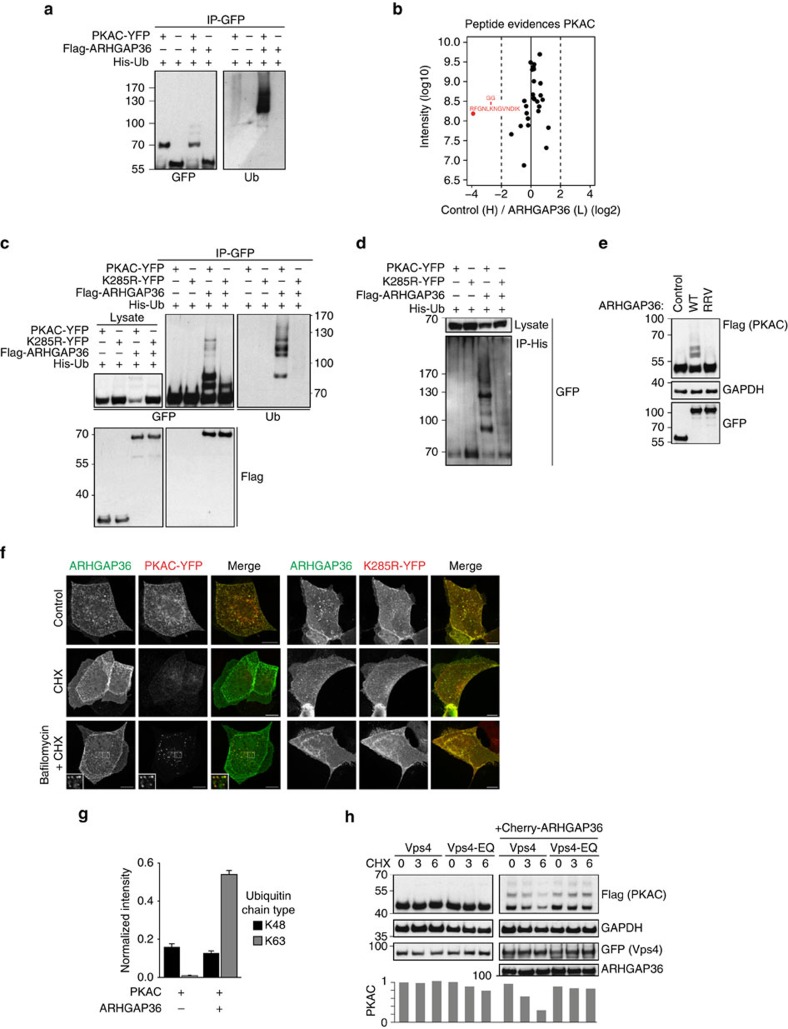
ARHGAP36 induces PKAC ubiquitylation and engagement with the ESCRT pathway. (**a**) HEK293T cells were transfected with PKAC-YFP or YFP-Cherry control, Flag-ARHGAP36 and His-Ubiquitin as indicated. Lysates were subjected to GFP IP. (**b**) SILAC-labelled HEK293T cells were transfected with PKAC-Flag in the presence or absence of YFP-ARHGAP36. Lysates were subjected to Flag IP. Normalized heavy/light ratio plot of the peptide evidences matching PRKAC. Only one peptide, highlighted in red, was upregulated in the presence of ARHGAP36 and was the only ubiquitylated PRKAC peptide identified. (**c**) The same as in **a** except where indicated PKAC-K285R-YFP was transfected and a Flag-Cherry control for Flag-ARHGAP36 was used. (**d**) The same as in **c** except lysates were subjected to His IP and immunoblotted with anti-GFP. (**e**) HEK293T cells were transfected with PKAC-Flag and CFP-ARHGAP36, the RRV mutant or CFP-Cherry control. Lysates were immunoblotted with the indicated antibodies. (**f**) Confocal micrographs of MDCK cells transfected with PKAC-YFP or PKAC-K285R-YFP and Flag-ARHGAP36. Twelve hours after transfection, cells were pretreated for 30 min with bafilomycin (100 nM), before cycloheximide (CHX; 1 μg ml^−1^) addition for a further 5 h. Cells were fixed and subjected to immunofluorescence using anti-GFP and anti-Flag. Scale bars, 10 μm. (**g**) HEK293T cells were transfected with PKAC-YFP in the presence or absence of Flag-ARHGAP36 and subjected to GFP IP followed by SRM-MS-based relative quantification of polyubiquitin linkage-type peptides. Data were normalized using the signal intensity of PKAC derived from a shotgun MS analysis of the same samples. Samples were measured in duplicate and the top two transitions used for quantification. The error bars represent the s.d. of the four calculated area ratios. (**h**) HEK293T cells were transfected with GFP-Vps4-WT or GFP-Vps4-EQ, PKAC-Flag and Cherry-ARHGAP36 or Cherry control as indicated. Cells were treated with CHX (50 μg ml^−1^) and collected at the indicated time points. Flag-PKAC levels were densitometrically evaluated and normalized to the first lane shown.

As ubiquitin is the key sorting signal for lysosomally directed cargos, we investigated whether this modification is required for ARHGAP36-dependent PKAC downregulation. Expressed on its own, PKAC-YFP is only minimally ubiquitylated. However, coexpression of Flag-ARHGAP36 strongly induced the appearance of higher-molecular-weight ubiquitylated species (Fig. 6a). Using SILAC-based MS, we identified a single ubiquitylation site on PKAC, K285, that was specifically regulated by ARHGAP36 (Fig. 6b; Supplementary Fig. 5a,b; Supplementary Data 1). Substitution to arginine, K285R, prevented ARHGAP36-induced polyubiquitylation and stabilized this mutant, while binding to ARHGAP36 was unaffected (Fig. 6c,d). A direct interaction with ARHGAP36 was essential for PKAC ubiquitylation, as the binding-deficient ARHGAP36-RRV mutant no longer induced ubiquitylation (Fig. 6e). Cycloheximide chase experiments revealed that both wild-type and K285R PKAC were stable for at least 9 h in the absence of ARHGAP36 (Supplementary Fig. 5c), in agreement with previous data^29^. However, in the presence of ARHGAP36, the protein half-life of wild-type PKAC was shorter than three hours, whereas the K285R mutant remained stable throughout. The degradation of wild-type PKAC could be rescued by bafilomycin, or the lysosomal protease inhibitor leupeptin, causing accumulation of PKAC on vesicles, whereas the K285R mutant was always stable (Fig. 6f; Supplementary Fig. 5d). These experiments demonstrate an absolute requirement of polyubiquitylation at K285 for ARHGAP36-mediated PKAC degradation.

The PKAC ubiquitylation pattern presented consistently with distinct high-molecular-weight bands (see Fig. 6c,d for example). While K48-polyubiquitylation typically targets proteins to the proteasome, K63-linked polyubiquitin chains are implicated in lysosomal degradation and binding to ESCRT-0 (Endosomal Sorting Complex Required for Transport)^30^,^31^,^32^,^33^. Selected reaction monitoring mass spectrometry (SRM-MS)^34^ identified a strong build-up in K63- but not K48-linked ubiquitin chains associated with immunoprecipited PKAC upon coexpression of ARHGAP36 (Fig. 6g). We also employed the UbICREST assay^35^, which makes use of deubiquitylase (DUB) cleavage specificity to identify chain linkages. We consistently detected partial cleavage by AMSH, a highly selective K63-linked ubiquitin chain editing endosomal DUB^36^, resulting in the accumulation of an additional lower-molecular-weight species corresponding to the expected end product of this reaction: mono-ubiquitylated PKAC. In contrast, the K48 linkage-specific DUB OTUB1 was without effect, while the whole ubiquitin smear was readily removed by the linkage-insensitive DUB USP2 (Supplementary Fig. 5e). These data indicate the involvement of K63-linked ubiquitin, and further support a model in which ARHGAP36 targets PKAC for lysosomal degradation.

Lysosomal targeting of ubiquitylated cargo relies on the ESCRT protein complexes. To test their involvement, we used a dominant-negative form of Vps4, an AAA-ATPase that is required for the recycling of ESCRT components and pinching off of intraluminal vesicles at a late step in the sorting mechanism^37^,^38^. Expression of wild-type Vps4 had no effect on ARHGAP36-induced PKAC degradation. However, overexpression of the dominant-negative E228Q mutant of Vps4 was able to rescue ARHGAP36-induced PKAC degradation, suggesting that this process is mediated by the ESCRT machinery (Fig. 6h). In summary, we conclude that ARHGAP36 targets PKAC for ubiquitin-dependent, ESCRT-mediated lysosomal degradation, a pathway usually reserved for membrane proteins.

### ARHGAP36 suppresses PKA signalling responses

**Figure 7:**
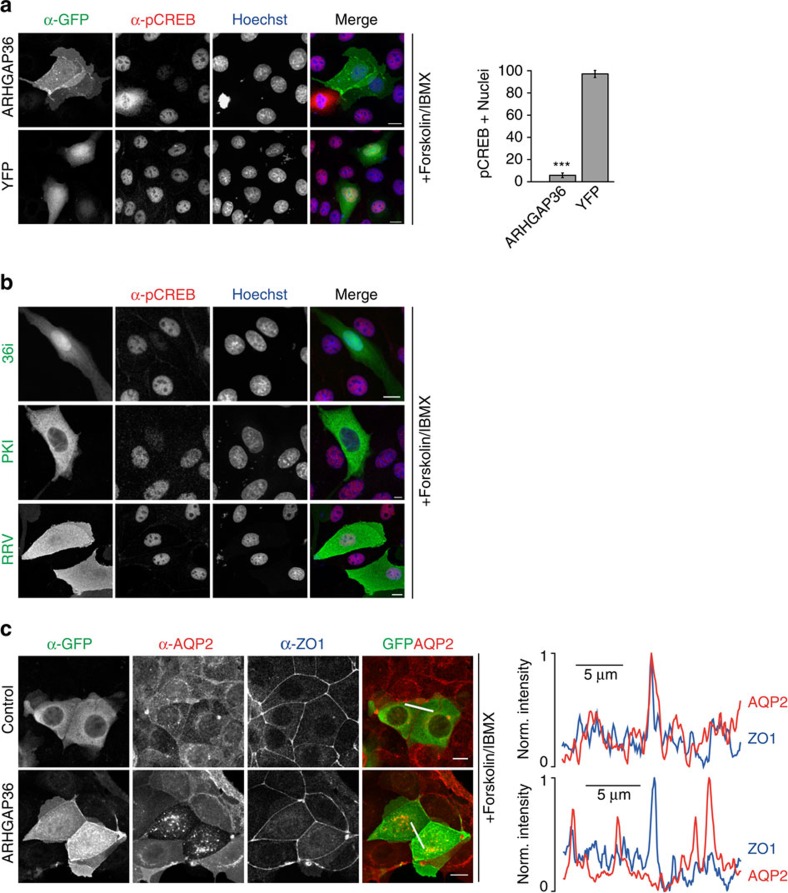
ARHGAP36 suppresses PKA signalling. (**a**) MDCK cells expressing YFP-ARHGAP36 or YFP control in low-serum conditions were treated with 10 μM Forskolin and 100 μM IBMX for 20 min, fixed and subjected to immunofluorescence using antibodies against GFP and phospho-CREB. Images were collected by confocal microscopy. Scale bars, 10 μm. Quantitative analysis of nuclear phospho-CREB staining in cells expressing the indicated constructs (*n*>100 for each of three independent experiments, shown as mean±s.e.m., ***P<0.001, Student's *T*-test). (**b**) As in **a**, except cells were transfected with Cherry-36i, CFP-PKI or CFP-ARHGAP36-RRV. (**c**) MCD4 cells stably expressing aquaporin-2 (AQP2) were transfected with YFP-ARHGAP36 or YFP control in low-serum conditions. Twelve hours post transfection, cells were treated with 10 μM Forskolin for 20 min. Fixed cells were subjected to immunofluorescence using antibodies against GFP, AQP2 and the tight junction protein ZO-1, to visualize the plasma membrane at cell–cell contact sites. Images were collected by confocal microscopy. Scale bars, 10 μm. Line scan fluorescence intensity profiles are shown on the right. In red: AQP2, in blue: ZO-1.

The above data suggest that ARHGAP36 strongly interferes with PKA signalling. Initiation of gene transcription on phosphorylation of the transcription factor cAMP response element-binding protein (CREB) is a well-characterized PKA-induced cellular response. Forskolin/IBMX stimulation of MDCK cells promoted phospho-CREB accumulation in the cell nuclei (Fig. 7a; Supplementary Fig. 6a). In contrast, in YFP-ARHGAP36-expressing cells, stimulation-dependent CREB phosphorylation was blocked. 36i expression also blocked CREB phosphorylation, as did full-length PKI, whereas ARHGAP36-RRV, the pseudosubstrate mutant, was ineffective (Fig. 7b and Supplementary Fig. 6b). As a second example of a PKA-dependent cellular process, we monitored the trafficking of the water channel aquaporin-2 (AQP2). In unstimulated kidney collecting duct cells, AQP2 localizes on vesicles. On cAMP elevation, AQP2 translocates to the plasma membrane, where it fine-tunes water homeostasis^39^. MCD4 mouse collecting duct cells, stably expressing human AQP2, were transfected with YFP-ARHGAP36 or YFP control. Without stimulation, AQP2 was located on vesicles (Supplementary Fig. 6c). On Forskolin stimulation, AQP2 translocated to the plasma membrane in control cells, whereas in YFP-ARHGAP36-transfected cells, AQP2 was retained on cytoplasmic vesicles (Fig. 7c). The above experiments show that PKA signalling is repressed in the presence of ARHGAP36.

### Endogenous ARHGAP36 antagonises PKAC in neuroblastoma cells

**Figure 8:**
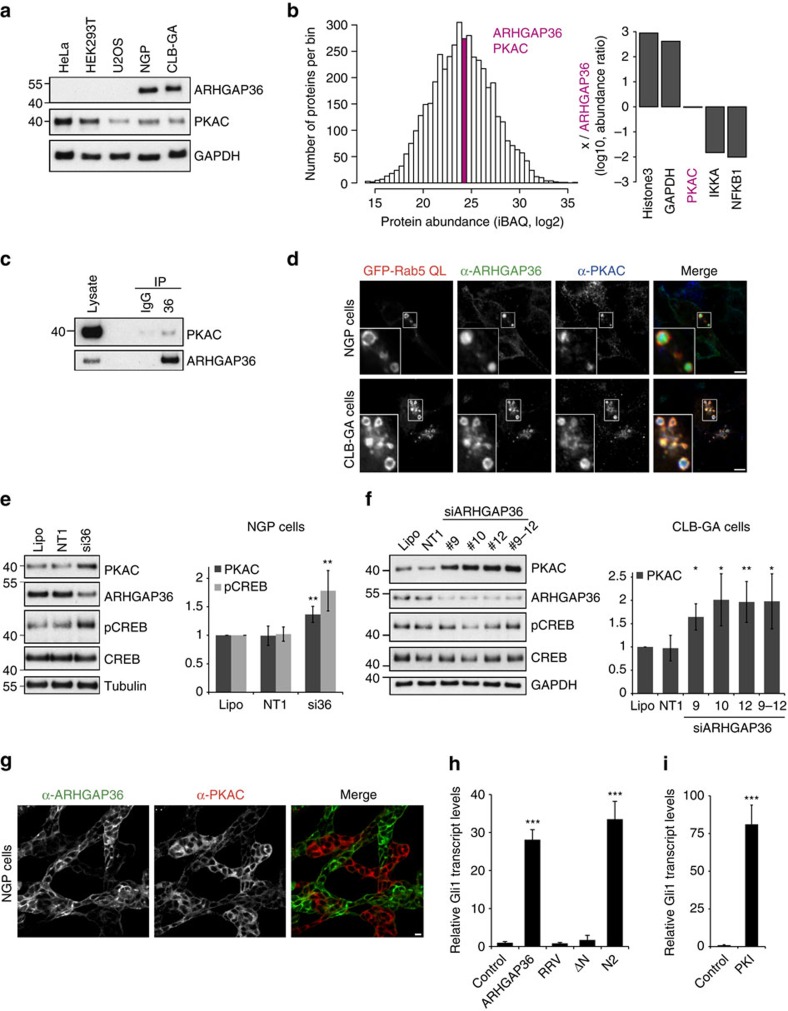
ARHGAP36 is expressed in neuroblastoma cells and promotes aberrant activation of the Hedgehog pathway. (**a**) An amount of 10 μg lysate of the indicated cell lines was immunoblotted with the indicated antibodies. ARHGAP36 is only present in NGP and CLB-GA neuroblastoma cells. The predominant isoform seems to be isoform 3, expected size 46 kDa. (**b**) Histogram displays the distribution of the relative abundance of all measured proteins (*n*=4448). ARHGAP36 and PKAC are within the same bin (pink). Bar plot shows the log 10 abundance of the indicated proteins relative to ARHGAP36, GAPDH (437-fold) and PKAC (0.95-fold). PKAC is the summed total of PRKACA and PRKACB. (**c**) NGP cell lysates were immunoprecipitated with an ARHGAP36 antibody or IgG rabbit control, and immunoblotted for ARHGAP36 or PKAC. 75% less lysate and eluate was run for ARHGAP36. (**d**) Confocal micrographs of NGP or CLB-GA cells transfected with GFP-Rab5-QL. Fixed cells were subjected to immunofluorescence using GFP, ARHGAP36 and PKAC antibodies. Scale bar, 5 μm. (**e**) Representative immunoblot of NGP cells treated with SMARTpool short interfering RNA (siRNA) against ARHGAP36 (si36) for 24 h. Cells were stimulated with 10 μM Forskolin and 100 μM IBMX for 30 min before collecting. Lysates were immunoblotted with the indicated antibodies. Lipo: reagent only control. NT1: non-targeting oligo control. Bands were densitometrically evaluated, normalized first to tubulin then to Lipo. Mean of five independent experiments±s.d. **P<0.01, Student's *T*-test compared with NT1. (**f**) Representative immunoblot of CLB-GA cells treated with individual or combined siRNA oligos against ARHGAP36 for 48 h. Bands were densitometrically evaluated, normalized first to tubulin then to Lipo. Mean of four independent experiments±s.d. **P<0.01 or *P<0.05, Student's *T*-test compared with NT1. (**g**) Confocal micrographs of NGP cells subjected to immunofluorescence using antibodies against ARHGAP36 and PKAC. Scale bar, 10 μm. (**h**) NIH3T3 cells transfected with the indicated CFP-tagged constructs for 15 h were serum starved for a further 48 h before collecting. Gli1 mRNA levels measured by quantitative PCR with reverse transcription were normalized to β-actin. Data shown as mean of three repeats±s.e.m., ***P<0.001, Student's *T*-test compared with control (**i**) As in **h**, except cells were transfected with CFP-PKI.

Next, we investigated whether ARHGAP36 also antagonises PKA in an endogenous setting. As we observed such a strong impact on PKA, we first wondered whether ARHGAP36 expression may be regulated during development or restricted to certain tissues. Indeed, mouse *in situ* hybridization revealed that *Arhgap36* is strongly expressed in skeletal muscle fibres during embryogenesis right up until birth, but is undetectable in postnatal muscles (Supplementary Fig. 7). This restricted tissue expression may explain the absence of ARHGAP36 in commonly used cell lines (Fig. 8a). However, expression databases suggested that ARHGAP36 is expressed in neuroblastoma cell lines (www.nextbio.com) and we indeed identified its expression at comparable levels in NGP and CLB-GA cells^40^,^41^, where endogenous ARHGAP36 localized similarly to the overexpressed protein (Fig. 8a; Supplementary Fig. 8a–c). Intensity-based absolute quantification (iBAQ) MS^29^ in NGP cells showed that ARHGAP36 and PKAC are expressed at approximately equimolar levels (Fig. 8b; Supplementary Fig. 8d). Furthermore, we could confirm that endogenous ARHGAP36 interacts with endogenous PKAC (Fig. 8c) and demonstrate that both proteins co-localize together on the limiting membrane as well as inside Rab5 Q79L enlarged endosomes (Fig. 8d). Importantly, acute knockdown of ARHGAP36 by RNA interference led to a significant increase in PKAC levels in both cell lines, and, in NGP cells, to a concomitant increase in CREB phosphorylation (Fig. 8e,f; Supplementary Fig. 8e,f). Moreover, immunofluorescence microscopy revealed a striking inverse correlation of ARHGAP36 and PKAC levels in the NGP cell line: cells with high ARHGAP36 expression exhibited low PKAC levels and vice versa (Fig. 8g; Supplementary Fig. 8g), presumably reflecting the existence of two sub-populations of cells in this non-clonal cell line. Collectively, these experiments demonstrate that ARHGAP36 both inhibits and degrades PKAC in an endogenous context.

### ARHGAP36 promotes aberrant activation of the Hh pathway

ARHGAP36 was recently reported to also be upregulated in a subset of medulloblastoma^18^, the most common malignant childhood brain tumour. This cancer is frequently associated with abnormal Hedgehog (Hh) signalling^42^, a key pathway controlling animal development^19^. ARHGAP36 was shown to activate Hh signalling in a Smoothened-independent manner^18^. However, how ARHGAP36 promotes oncogenic Hh activation was unknown. We thus asked whether ARHGAP36 activates this pathway via its effects on PKA. Using NIH3T3 cells, we monitored the transcript levels of endogenous Gli1, a Hh target gene commonly used as a readout for pathway activity^43^. We first confirmed that these cells were responsive to Hh stimuli (Supplementary Fig. 8h) and then assessed the ability of different ARHGAP36 constructs to activate the Hh pathway in the absence of ligand. In agreement with the previous data^18^, we observed a robust increase in Gli1 transcripts in ARHGAP36-expressing cells (Fig. 8h). ARHGAP36-N2, containing the pseudosubstrate motif, but not ARHGAP36-ΔN, lacking this motif, also promoted Gli1 expression. Importantly, the ARHGAP36-RRV mutant that is unable to bind and inhibit PKAC was without effect. We also observed a strong increase in Gli1 transcripts on overexpression of PKI, showing that PKA inhibition can indeed induce the Hh pathway in a ligand-independent manner (Fig. 8i). We conclude that ARHGAP36 promotes Hh signalling by suppressing PKAC, underpinning the role of PKA as a negative regulator of the Gli proteins^19^,^20^,^44^. Our experiments thereby provide a mechanism by which aberrant ARHGAP36 expression may promote medulloblastoma formation. Our data raise the possibility that abnormal ARHGAP36 expression could contribute to further disease settings that are regulated by PKA signalling.

## Discussion

We here report the identification of ARHGAP36 as a potent PKA antagonist, employing two different inhibitory mechanisms. Peptide spot mapping, point mutagenesis, ITC and activity assays revealed that ARHGAP36 directly binds to PKAC via a pseudosubstrate motif by which it blocks PKA activity with potency comparable to the PKI proteins. In addition, it mediates the ubiquitylation of PKAC and targets it for endolysosomal degradation. As a consequence, PKA signalling is suppressed in the presence of ARHGAP36. This is the first report of ubiquitylation-dependent downregulation of PKAC. Many other kinases are known to be subject to regulated proteolysis as an efficient means of signal termination^14^,^15^. To our surprise, ARHGAP36 targets PKAC not to the proteasome, the default pathway for the downregulation of cytosolic proteins, but for endolysosomal degradation. This pathway typically processes endocytosed transmembrane receptors, such as the epidermal growth factor receptor^14^,^15^,^45^.

To our knowledge, only one other cytosolic kinase, GSK3, has been reported to be incorporated into the endolysosomal system in an ESCRT-dependent manner^46^. In response to sustained Wnt stimulus, GSK3 is sequestered into MVBs away from its cytosolic substrates, in particular ß-catenin, which is otherwise constitutively turned over. However, both the mechanism by which GSK3 is targeted to MVBs and its subsequent fate are unclear. In contrast to PKAC in the present study, GSK3 has not been reported to be ubiquitylated and thus the mechanism by which GSK3 engages with the ESCRT machinery remains unresolved. ARHGAP36 appears to be partially incorporated into the lumen of Rab5 Q79L enlarged endosomes together with PKAC. The endosomal E3 ligase and multi-subunit adaptor protein CBL has also been seen to translocate with cargo into the internal vesicles of MVBs^47^. However, the bulk of overexpressed ARHGAP36 does not seem to be degraded, as total protein levels do not change, even on cycloheximide treatment (Fig. 6f,h; Supplementary Fig. 5c,d). While we have so far failed to identify the E3 ligase mediating PKAC ubiquitylation, we show the strict requirement of a single ubiquitylation site (K285) in the Vps4-dependent incorporation of PKAC into MVBs and its subsequent rapid and efficient degradation in lysosomes. Interestingly, GSK3 is also subject to a bimodal negative regulation in that its kinase activity is initially inhibited by binding to the intracellular domain of the LRP6 receptor on Wnt stimulation^48^,^49^. Our work raises the question whether endolysosomal degradation of cytosolic signalling proteins represents a more general mechanism for effective long-term signal termination. Entry into this pathway may be facilitated by a membrane-associated interaction partner that can mediate access to endocytic sorting.

In contrast to PKAC, PKAR has been shown to be degraded by the proteasome, during the establishment of long-term synaptic plasticity. The resultant change in PKAR/PKAC ratios causes persistent PKAC signalling that is uncoupled from cAMP levels^12^,^13^, the spatial separation of PKAR and PKAC degradation may be important in such a mechanism. It is possible that PKAC is less amenable to unfolding by the AAA-ATPases of the proteasomal regulatory particle, which is a prerequisite for entry into the core particle and thus degradation^50^. Alternatively, ARHGAP36-mediated endolysosomal degradation of PKAC may provide a mechanism for timely inactivation and selective degradation of a restricted pool of PKAC, thus contributing to the compartmentalization of PKA signalling responses. Localized regulation of PKAC may also explain why the phosphorylation status of the nuclear substrate CREB is unchanged on depletion of ARHGAP36 in CLB-GA cells: in these cells ARHGAP36 is predominantly localized to endosomal membranes.

ARHGAP36 further extends the repertoire of PKA regulators. Whereas modulation of PKA enzymatic activity is generally centred on PKAR, ARHGAP36 directly binds to PKAC and uncouples it from upstream control via PKAR. The dual-mode PKAC inhibition by ARHGAP36, pseudosubstrate binding and subsequent degradation, ensures both immediate and sustained negative control of PKA. Dependent on the context this could operate in a constitutive manner: ARHGAP36 may serve as a tonic suppressor of PKA to profoundly dampen the sensitivity of certain tissues to cAMP-releasing stimuli. It could also oppose basal PKA activation through cAMP-independent mechanisms in the absence of stimulus, particularly in settings with stoichiometric excess of free PKAC. Compensatory mechanisms have been proposed to balance the relative PKAR/PKAC expression levels to preserve proper responsiveness of the PKA holoenzyme to cAMP^51^,^52^. ARHGAP36 may participate in such a regulation by providing additional buffering capacity.

We observed temporally restricted expression of *Arhgap36* in skeletal muscles during mouse embryonic development. Fittingly, expression profiling identified *Arhgap36* (RIKEN cDNA 1100001E04) as a major target gene of the myogenic transcription factor MyoG^53^. ARHGAP36 may also antagonise PKA in other select tissues. It is tempting to speculate that it is expressed in contexts where additional safeguard mechanisms are needed to ensure tight control of PKA. Future gene targeting studies in mice will reveal the details of such tissue-specific PKA desensitization and thus the role of ARHGAP36 in development and homeostasis.

Since ARHGAP36 is a strong suppressor of PKA signalling, it is conceivable that aberrant ARHGAP36 expression is implicated in disease. It will be interesting to further explore the role of ARHGAP36 and PKA in neuroblastoma. In human medulloblastoma, upregulated ARHGAP36 has been linked to its ability to activate Hh signalling in a Smoothened-independent manner^18^. Our work strongly suggests that this ARHGAP36-dependent oncogenic pathway activation is via inhibition of PKAC. ARHGAP36 is also found to be upregulated in human pheochromocytomas, a neuroendocrine tumour arising from chromaffin cells of the adrenal medulla^54^. Deregulated PKA signalling due to mutations in the cAMP/PKA axis is associated with numerous other diseases, such as Cushing's syndrome, hepatocellular carcinoma, basal cell carcinoma, Carney complex and McCune–Albright syndrome^2^,^3^,^4^,^5^,^6^,^7^,^8^,^55^,^56^. Dependent on the signalling context in which PKA functions, aberrant ARHGAP36 expression could have both tumour suppressive and oncogenic roles.

In conclusion, our data indicate that ARHGAP36-mediated PKAC inhibition and downregulation is a potent shutdown mechanism to efficiently suppress PKA-dependent cellular responses. This mechanism involves the unexpected uptake of cytosolic PKAC into the endolysosomal system for subsequent degradation. Our work extends the molecular map of the regulatory circuitry controlling the highly conserved cAMP signalling pathway and provides new insights into its dysregulation in human disease.

## Methods

### Cell lines

Human embryonic kidney (HEK293T) cells were cultured in DMEM containing 10% fetal bovine serum (FBS) and transfected using polyethylenimine (PEI). MDCK cells were cultured in MEM containing 10% FBS and transfected using Effectene (Qiagen). MCD4 mouse collecting duct cells stably expressing human AQP2 (refs 39, 57) were cultured in DMEM/F12 supplemented with 5% FBS and 5 μM dexamethasone and transfected using PEI. NIH3T3 mouse fibroblasts and U2OS human osteosarcoma cells were cultured in DMEM containing 10% FBS and transfected using Lipofectamine 3000. NGP and CLB-GA cells were cultured in RPMI containing 10% FBS and transfected using Lipofectamine 3000.

### Chemicals

Forskolin, IBMX, bafilomycin and epoxomicin were purchased from Cayman, Leupeptin from US Biological, cycloheximide from Calbiochem, SAG (Smoothened AGonist) from Enzo and CellTracker Deep Red from Thermo.

### Plasmids

cDNAs for overexpression of ARHGAP36 were subcloned into plasmids of the Creator system described previously^58^. The full-length sequence refers to human ARHGAP36 UniProt ID: Q6ZRI8-2. Subsequent mutation and deletion constructs were created by site-directed mutagenesis (Agilent Technologies). 36i was amplified with the following primers: 36i_N1_f 5′-gcgcggaattcgccaccatggagcccaccttgccccgg-3′ and 36i_N1_r 5′-cgcgcaccggtccgctcagctcagccagactatccac-3′, or 36i_C1_f 5′-gcgcgtgtacagcgagcccaccttgccccgg-3′ and 36i_C1_r 5′-cgcgcgaattcctacagctcagccagactatc-3′, and inserted into pmCherry-C1 (Clontech) with BsrGI and EcoRI or into pmCherry-N1 (Clontech) with EcoRI and AgeI. Mouse PKAC-Venus-YFP was provided by Manuela Zaccolo (University of Oxford)^59^. Human PRKACA (GenBank: BC039846.1) was cloned into a C-terminal Flag-tagged gateway vector (Life Technologies). K285R mutants were generated by site-directed mutagenesis. Human PRKAR1α (GenBank: BC036285) was cloned into the gateway system. Cerulean CFP-PKI was kindly provided by Susan Taylor (University of California, San Diego). AKAR4-NES and PKI-Cherry were provided by Jin Zhang (Johns Hopkins)^26^,^60^. LAMP1-YFP was a gift from Lee Haynes (University of Liverpool). GFP-HRS was described previously^61^. GFP-Vps4 and the EQ mutant were a gift from Phil Woodman (University of Manchester)^37^ and GFP-Rab5 Q79L was a gift from Volker Haucke (FMP, Berlin)^62^.

### Lysis and immunoprecipitation and western blotting

HEK293T cell pellets were lysed in NP40 lysis buffer (1% NP40, 20 mM Tris-HCL pH 7.5, 150 mM NaCl, 1 mM EGTA, 5 mM NaF) with complete protease inhibitor cocktail (Roche) and *N*-ethylmaleimide (NEM, Sigma). For immunoprecipitation (IP), cleared lysates were added to either Flag-M2 affinity gel (Sigma) or Protein G Sepharose beads (Sigma) coupled with anti-GFP (ab290, Abcam, 0.5 μl per 10 cm plate) or anti-His (H1029, Sigma, 1 μl per 10 cm plate). After minimum 1 h rotation at 4 °C, beads were washed three times in lysis buffer and eluted with Laemmli buffer. NGP and CLB-GA cells were lysed directly in RIPA (50 mM Tris pH 7.4, 150 mM NaCl, 0.1% SDS, 1% NP40, 0.5% sodiumdeoxycholate). For normal lysis, RIPA was supplemented with complete protease inhibitor cocktail (Roche), NEM and PhosSTOP (Roche). For IP experiments, RIPA was supplemented with EDTA-free complete protease inhibitor cocktail (Roche), NEM, 5 mM MgCl_2_ and 1 mM ADP. An amount of 4.5 mg protein was incubated for 1 h at 4 °C with 4 μg anti-ARHGAP36 (Thermo, PA5-31619) antibody or IgG control. Beads were then added for 30 min, washed three times in lysis buffer and eluted with Laemmli buffer. The following antibodies were used for western blotting: phospho-CREB (CST, 9198, 1:1,000), CREB (CST, 9104, 1;1,000) GAPDH (CST, 2118, 1:5,000), Flag (Sigma, M2, 1:5,000), His (Sigma, H1029, 1:500), Tubulin (Sigma, DM1a, 1:10,000), PKAC (BD, 610981, 1:1,000), GFP (Abcam, ab290, 1:10,000), Ubiquitin (Covance, P4G7, 1:500), ARHGAP36 (Atlas, HPA002064, 1:500), RIα (BD, 610610, 1:1000), RIβ (R&D, AF4177, 1:500), RIIα (BD, 612243, 1:1,000), RIIβ (BD, 610626, 1:1,000), β-catenin (BD, 610153, 1:5,000). All uncropped blots are shown in Supplementary Figs 9 and 10.

### Short interfering RNA knockdown

NGP cells were cultured for 2 days, CLB-GA for one, before transfection with 50 nM ARHGAP36 SMARTpool or individual oligos, or non-targeting oligo 1 (NT1) (Dharmacon) using Lipofectamine RNAiMAX (Invitrogen) according to the manufacturer's guidelines. Twenty-four or 48 h later, cells were lysed directly in RIPA. Where indicated, cells were stimulated with 10 μM Forskolin and 100 μM IBMX for 30 min before collecting. ARHGAP36 On Target Plus Oligos: J-032590-12, 5′-GCGGGUCAGCUCCGAGAAA-3′; J-032590-11, 5′-CCUCGGAGACGGACAUCGA-3′; J-032590-10, 5′-UAUGAGAUUUACCGGGAUU-3′; and J-032590-09, 5′-GCAUGCAGAGAGAGCGCUA-3′.

### Immunoflourescence

Cells seeded on glass coverslips were fixed with 4% paraformaldehyde for 10 min, permeabilized with 0.2% Triton X-100/100 mM glycine for 10 min and blocked for 20 min in 3% bovine serum albumin, before primary antibody incubation for 1 h at room temperature or at 4 °C overnight. Alexa Fluor secondary antibodies (Molecular Probes, 1:1,000) were incubated for 30 min. Coverslips were mounted using ProLong Gold (Invitrogen). The following antibodies were used: phospho-CREB (CST, 9198, 1:200), EEA1 (CST, 3288, 1:200), Flag (Sigma, M2, 1:500), PKAC (BD, 610981, 1:200), GFP (Abcam, ab13970, 1:1,000), ZO-1 (Santa Cruz, sc33725, 1:1,000) ARHGAP36 (Atlas, HPA002064, 1:100), RIIα (Santa Cruz, sc-909, 1:200) and RIIβ (BD, 610626, 1;200). Rabbit polyclonal anti-HRS (1:500) and rabbit polyclonal anti-AQP2 (H27, 1:1,000) were custom made^61^,^63^.

### Peptide spots

Peptide spots were produced by automatic SPOT synthesis on Whatman 50 cellulose membranes using Fmoc (9-fluorenylmethyloxycarbonyl) chemistry with the AutoSpot-Robot ResPep-SL (Intavis Bioanalytical Instruments) as described previously^64^. Fmoc-protected amino acids and derivatized cellulose membranes (amino-modified acid-stable cellulose membrane with polyethylene glycol spacer) were purchased from Intavis. Membranes were incubated with 0.1 μg μl^−1^ GST-PKAC (Biovision) and interactions were detected using anti-GST (CST, 26H1, 1:5,000), as for western blotting.

### UbiCREST assay

The assay was purchased from Boston Biochem and carried out according to the manufacturer's guidelines and as described previously^35^. Specifically, HEK293T cells were transiently transfected with PKAC-YFP, Flag-ARHGAP36 and His-Ubiquitin. Cells were lysed in the presence of NEM. Lysates were subjected to one single GFP IP, which after washing, was then split and incubated with the DUBs for 45 min at 37 °C. Eluates were run on 12% SDS–polyacrylamide gel electrophoresis gels, and membranes were probed with GFP and ubiquitin antibodies.

### Fluorescence microscopy

Confocal laser scanning microscopy was performed on a Fluoview 1000 confocal microscope (Olympus) equipped with a UPLSAPO × 60/1.3 numerical aperture silicon immersion oil immersion lens. Images were taken with the following excitation (Ex) and emission (Em) settings: Hoechst Ex: 405 nm diode laser (50 mW) Em: 425–475 nm; mCerulean-CFP Ex: 440 nm diode laser (25 mW) Em: 460–500 nm; GFP, AlexaFluor488 Ex: Multi-Line Argon laser 488 nm (40 mW) Em: 500–545 nm; mCitrine-YFP, Venus-YFP Ex: Multi-Line Argon laser 515 nm (40 mW) Em: 530–545 nm; AlexaFluor555 Ex: 559 nm diode laser (20 mW) Em: 570–625 nm; mCherry Ex: 559 nm diode laser (20 mW) Em: 575–675 nm; AlexaFluor647 Ex: 635 nm diode laser (20 mW) Em: 655–755 nm.

### Time-domain FLIM

Time-correlated single photon counting measurements to acquire fluorescence lifetime images were carried out at room temperature using the Fluoview 1000 confocal microscope equipped with a FLIM Upgrade kit (Picoquant). Venus-YFP was excited with a 485 nm diode laser (LDH-D-C-485, Picoquant) at 40 MHz repetition, spectrally filtered using a band emission filter (HQ 550/49, AHF, Germany), and fluorescence was collected through a silicone immersion oil objective ( × 60/1.3 UPLSAPO, Olympus). Photons were detected using a single photon avalanche photodiode (1 channel LSM-SPAD, Picoquant) and timed using a single photon counting module (PicoHarp 300, Picoquant). FLIM data were background subtracted and processed using the pixel-based fitting software SymPhoTime 64 (Picoquant) to calculate the lifetime maps, histograms and average lifetimes of Venus-YFP. Goodness of fit was assessed by the calculated standard least squares (*χ*^2^) and a mono- or bi-exponential fitting model was applied accordingly.

### AKAR FRET

AKAR4-NES is a monomolecular ratiometric FRET sensor containing a PKA substrate site and a FHA1 phosphoamino acid-binding domain sandwiched between Cerulean CFP donor and cpVenus acceptor fluorophores and can thus be used to probe relative PKA activity levels in living cells^26^,^60^. Intensity-based FRET ratio imaging experiments were performed in HEK293T cells on 24-well plates (μ-plate Ibidi) on an inverted epifluorescence IX81 microscope (Olympus) equipped with a MT20 xenon-arc burner (Olympus) and an UPLSAPO air × 10/0.4 numerical aperture objective controlled by xcellence software (Olympus). Images were acquired with a Hamamatsu ImagEM Enhanced EM-CCD camera at a 16-bit depth. Donor and FRET acceptor images were acquired with the following settings: 430/25 (excitation), zt442RDC (dichroic mirror) and emission changing between 483/32 (donor channel) and 542/27 (FRET acceptor channel) using a filter wheel. Images were analysed with ImageJ. Images were background corrected and regions of interest were defined using the AKAR4-Venus fluorescence channel. Average intensities of the acceptor channel were divided by the donor channel to to calculate the FRET ratio.

### PepTag PKA activity assay

PepTag Assay (Promega) was carried out as described previously^65^. Specifically, A1 Peptide (L-R-R-A-S-L-G) was incubated with or without 25 ng recombinant PKACα (Biovision) together with 10 μM scrambled, 36i or PKI (5–24) peptides for 45 min on a shaker at 30 °C, before separation of phosphorylated and non-phosphorylated peptide by agarose gel (0.8%) electrophoresis. PKI (5–24) peptide for this assay was purchased from Santa Cruz (TTYADFIASGRTGRRNAIHD).

### Peptide Synthesis

Peptides were synthesized on a 433A peptide synthesizer (Applied Biosystems) on Rapp resin columns (Rapp Polymere, Tuebingen, Germany) using Fmoc (**N**-(9-fluorenyl)methoxycarbonyl) chemistry. The peptides were purified and analysed using high-performance liquid chromatography on Polyencap A300 columns (Bischoff, Leonberg, Germany) and electrospray MS (TSQ 700, Finnigan MAT, Bremen, Germany).The sequences of the peptides were

36i: E-P-T-L-P-R-E-F-T-R-R-G-R-R-G-A-V-S-V-D-S-L-A-E-L

Scrambled: V-R-R-F-P-T-R-A-E-E-T-S-L-E-G-A-G-R-S-R-V-L-L-P-D

PKI (5–24): T-T-Y-A-D-F-I-A-S-G-R-T-G-R-R-N-A-I-H-D

### Hedgehog signalling assay

NIH3T3 cells seeded on 24-well plates were transfected with 500 ng plasmid for 16 h. The following day, the medium was replaced with starvation medium (0.2% FCS) and cells were cultured for a further 48 h. For control experiments shown in Supplementary Fig. S8h, NIH3T3 cells were cultured for 24 h in the presence of 0.2 μM SAG or conditioned medium (collected from HEK293 cells stably secreting SHH-Np (SHHN-293 cells kindly provided by M. Kato, Stanford School of Medicine)) at a 1:10 dilution in medium containing 0.5% FCS. RNA was isolated using TRIzol reagent (Life Technologies) and transcribed to cDNA using the RevertAidFirst Strand cDNA Synthesis kit (Thermo Scientific). Quantitative PCR was performed using Maxima SYBR Green qPCR Master Mix (Thermo Scientific) and a Bio-Rad iQ5 Thermal Cycler. cDNA was amplified using the following primers: Gli1_fw: 5′-CCAGCAGCTGCACTGAAGGATC-3′, Gli1_rv: 5′-CCAGCTCTGACTTCAGCTGG-3′, mβ-actin_fw: 5′-GTCCACACCCGCCACCAGTTC-3′, mβ-actin_rv: 5′-GGCCTCGTCACCCACATAG-3′.

### MS-based ubiquitin site identification

For ubiquitin site identification, HEK293T cells were fully labelled in stable isotope labelling by amino acids in cell culture (SILAC) media, heavy or light, before being transiently transfected with PEI. Cells were collected after 24 h, lysed by sonicating in RIPA buffer (50 mM Tris-HCl, pH 7.4, 150 mM NaCl, 0.25% Na-deoxycholate, 1 mM EDTA) containing 0.1% SDS and supplemented with NEM, DNAse and MPI. Lysates were subjected to IP using Flag-M2 affinity gel (Sigma). Heavy and light corresponding samples were mixed before the last washing step. Proteins eluted in guanidine-HCl were precipitated in ethanol over night at 4 °C as previously described^66^. Proteins were spun down and ethanol decanted. Protein pellet was resuspended in 6 M urea and 2 M thiourea in 10 mM HEPES (pH 8). Proteins were denatured using dithiothreitol followed by alkylation of cysteines by chloroacetamide. Proteins were digested by endoproteinase LysC and then diluted in 50 mM ammoniumbicarbonate (pH 8) in water before being further digested by trypsin over night at room temperature. Resulting peptide solution was desalted on stage tips by washing in 5% acetonitrile and 0.1% formic acid^67^. Samples were eluted in 80% acetonitrile and 0.1% formic acid and vacuum dried before being diluted in 5% acetonitrile and 0.1% formic acid. The peptides were separated on a 15 cm column packed in house with ReproSil-Pur 120 C18-AQ 3 μm resin (Dr Maisch GmbH), using a 1 h linear gradient of increasing acetonitrile concentration with a flow rate of 250 nl min^−1^ on a high-pressure liquid chromatography system (ThermoScientific). Separated peptides were ionized using an electrospray ionization source (ThermoScientific) and analysed on a Q-Exactive mass spectrometer (ThermoScientific). The Orbitrap resolution was set to 70,000 (target value 3,000,000; maximum injection time of 20 ms) for full scans and 17,500 (maximum injection time 60 ms; target value 1,000,000) for tandem mass spectrometry (MS/MS) spectra. The system was run in a data-dependent mode selecting the top 10 most intense ions for higher-energy collision-induced dissociation.

Raw files were analysed using the MaxQuant software 1.5.1.1 (ref. 68) using the default setting but with ‘Requantify' and ‘match between runs' activated. Lys8 and Arg10 were set as the heavy label. Ubiquitin leaves a signature GlyGly modification of Lysines (+114.0429 Da) after tryptic digestion. The GlyGly(K) modification, acetylation of protein N-termini and oxidation of methionine were set as variable modifications. C-terminal carbamidomethylation was set as a fixed modification. Trypsin/P was set as protease for the *in silico* digest of the Human Uniprot database (2014-01) in addition to a database containing common contaminants. The false discovery rate was set to 1% both at the peptide and protein level, and was estimated by in parallel matching the MS/MS spectra against a database containing the reversed sequences of the Uniprot database^68^.

Plotting of the SILAC ratios was done using R version 2.15.1 (R Foundation for Statistical Computing, Vienna, Austria) and spectra were visualized by the MaxQuant viewer. Both figures were modified in Illustrator (Adobe).

### SRM chain linkage identification

For SRM chain linkage identification, HEK293T cells were lysed as described and subjected to IP using GFP-Trap Beads (Chromotek) before washing with 2 M guanidine-HCl, followed by water and eluting in 2 × SDS sample buffer. Eluates were run briefly on an SDS–polyacrylamide gel electrophoresis gel followed by an in-gel digestion with trypsin. After a solid-phase extraction and desalting, peptides were eluted, lyophilized and reconstituted with a 0.1% formic acid/3% ACN buffer containing 100 fmol μl^−1^ of heavy tryptic peptide standards corresponding to all the different polyubiquitin linkage types^34^. Peptides were separated on a reversed-phase column (20 cm length, 75 μm ID, 3 μm Dr Maisch C18) with a gradient from 3 to 36% ACN in 38 min and SRM measurements performed using a Q-Trap 6500 (AB Sciex). The top two most intense transitions were selected and their peaks integrated with MultiQuant 3.0 software (AB Sciex). In parallel, the samples were measured in data-dependent acquisition mode using an Orbitrap Q-Exactive instrument (Thermo) with an on-line chromatography equivalent to what was described for the SRM analysis. The raw files were analysed with MaxQuant 1.5.2.8 and the signal intensity for PKAC used to normalize the SRM-MS data to account for variation between the IPs.

### Global protein abundance measurements

For iBAQ, NGP cells were collected, pelleted and flash-frozen before precipitation of proteins with equal volumes of chloroform, methanol and water^69^. Proteins were solubilized in 6 M urea/2 M thiourea buffer containing 10 mM HEPES and treated with benzonase to digest DNA and reduce viscosity. An amount of 50 μg lysate was subjected to reduction with TCEP (tris(2-carboxyethyl)phosphine) and alkylation with CAA (2-chloroacetamide) before digestion with trypsin. Peptides were extracted and desalted with a C18 StageTip before elution and reconstitution in 3% ACN/0.1% formic acid, followed by separation on a reversed-phase column (20 cm length, 75 μm ID, 3 μm Dr Maisch C18) with a gradient from 5 to 45% ACN in 120 min, while MS and MS/MS spectra were acquired in data-dependent mode on a Q-Exactive Plus instrument (Thermo). Raw data files were analysed with MaxQuant 1.5.2.8.

To calculate protein abundances, we applied the ‘iBAQ' algorithm^29^. In short, we first calculated the number of theoretically observable peptides per protein by *in silico* trypsin digesting the Uniprot data base. We then divided the protein intensities by the theoretical observable peptide count.

### Protein expression and purification

Human wild-type PKAC was expressed as an N-terminal His-tag fusion in *Escherichia coli* BL21 (DE3) Rosetta (Novagen) from a pET46 Vector. Bacteria cultures in LB medium were induced at an optical density of 0.6 with 40 μM isopropyl-β-D-thiogalactoside, grown overnight at 18 °C and lysed in 50 mM HEPES (pH 7.5), 300 mM NaCl, 20 mM imidazole, 5 mM MgCl_2_ and 2.5 mM ß-mercaptoethanol using a microfluidizer (Microfluidics). After centrifugation at 40,000*g* for 45 min at 4 °C, the soluble extract was filtered and applied to a Ni-NTA column (GE-Healthcare). The column was washed with the above buffer, containing 40 mM imidazole. His-PKAC was eluted in buffer containing 100 mM imidazole. PKAC was further purified by size-exclusion chromatography on a Superdex S75 26/60 column (GE) equilibrated with 50 mM HEPES (pH 7.5), 150 mM NaCl and 5 mM MgCl_2_. Monomeric His-PKAC eluted as a symmetrical peak that was pooled, concentrated to ∼50 mg ml^−1^ and snap-frozen in liquid nitrogen until further use for ITC experiments. The yield was 2.3 mg purified PKAC protein per 1 l bacteria culture.

### ITC measurements

Peptide stock solutions were prepared in the same buffer used for the final purification step of His-PKACα (50 mM HEPES, pH 7.5, 150 mM NaCl, 5 mM MgCl_2_). Peptide concentration of PKI and 36i was determined with the Direct Detect Spectrophotometer (EMD Millipore). Quantification by absorbance at 280 nm was not possible, as 36i does not contain tyrosine or tryptophan residues. Peptide concentrations were determined from triplicate measurements using 2 μl of diluted (ca. 2 mg ml^−1^) peptide solution with the above mentioned buffer used as a blank. ITC data were acquired on a microcalorimeter VP-ITC (MicroCal Inc). Stock solutions of PKAC, PKI and 36i were dissolved in 50 mM HEPES, pH 7.5, containing 150 mM NaCl, 5 mM MgCl_2_ and 2 mM ATP analogue adenylyl-imidodiphosphate. Titrations were conducted at 25 °C using 30 μM PKAC and 507 μM PKI or 351 μM 36i, respectively. Samples were stirred at 351 r.p.m. Injections were separated by 280 s of equilibration. A one-site binding model was assumed and the data were fitted using Microcal Origin software (version 7.0).

### Image analysis

Line scans to determine fluorescence intensity profiles were analysed using ImageJ software. In brief, plot profile analysis was used to create a graph of pixel intensity plotted against the distance along the line. To reduce noise, pixel intensity was averaged on a linewidth of 10 pixels. Intensity was then normalized to the lowest and the highest intensity within the selection. For analysis of protein level ratios of ARHGAP36 and PKAC in immunofluorescence images of NGP cells, automated cell segmentation was performed with CellProfiler software^70^ based on 4,6-diamidino-2-phenylindole (DAPI), CellTracker Deep Red and ARHGAP36 images. Average PKAC and ARHGAP36 intensities were measured in each cell outline. Each single-cell intensity was normalized to the median intensity of all cells in each image. Pearson's sample correlation analysis was performed in OriginPro (OriginLab).

### *In situ* hybridization

Specific fragments of the Arhgap36 gene were amplified from E12.5 whole mouse embryo cDNA using the following primers: 5′-GCATCTGTCAATGTTGTCCG-3′ and 5′-GGTGGCAAATTTGCCCTTCTTCC-3′. The PCR product was cloned into pGEM-T Easy plasmid using T4 DNA ligase (Promega). *In vitro* transcription of the antisense probe was performed using the DIG-RNA labelling kit (Roche). Paraffin sections were rehydrated by successive incubations in decreasing concentrations of ethanol and additional incubation in xylol. Subsequently, the sections were postfixed in 4% paraformaldehyde for 20 min and incubated in 10 μg ml^−1^ Proteinase K solution for 8 min. The tissue was hybridized with the RNA probe overnight at 65 °C. Following washings with SSC/formamide and MABT, the sections were incubated in blocking reagent (Roche) and goat serum. The section was then incubated with an alkaline-phosphatase-coupled antibody against digoxigenin (Roche). On washing with MABT and NTMT, NBT/BCIP Purple (Roche) was used as a chromogenic substrate for the alkaline phosphatase (Boehringer) to detect the RNA probe. The sections were mounted with Roti-Histokitt II (Roth) and imaged on a Leica DMI 6000B inverted microscope.

### Statistics

An unpaired Student's *T*-test was used to evaluate statistical significance. Values are expressed as the mean±s.e.m. or s.d. as indicated. Significance was set at the 95% confidence level and ranked as *P<0.05, **P<0.01, ***P<0.001.

### Data availability

Data supporting the findings of this study are available within the article and its Supplementary Information files and from the corresponding author on reasonable request.

## Additional information

**How to cite this article**: Eccles, R. L. *et al*. Bimodal antagonism of PKA signalling by ARHGAP36. *Nat. Commun.* **7**, 12963 doi: 10.1038/ncomms12963 (2016).

## Supplementary Material

Supplementary Information Supplementary Figures 1-10 and Supplementary References

Supplementary Data 1 SILAC mass spectrometry identification of K285 ubiquitin on PKAC-Flag
