# Supplementary material for: Bimodal antagonism of PKA signalling by ARHGAP36
Source: Nat Commun. 2016 Oct 7;7:12963. doi: 10.1038/ncomms12963 (PMC5059767; doi:10.1038/ncomms12963)
Supplement: Supplementary Information — Supplementary Figures 1-10 and Supplementary References [file ncomms12963-s1.pdf]

Supplementary Figure 1

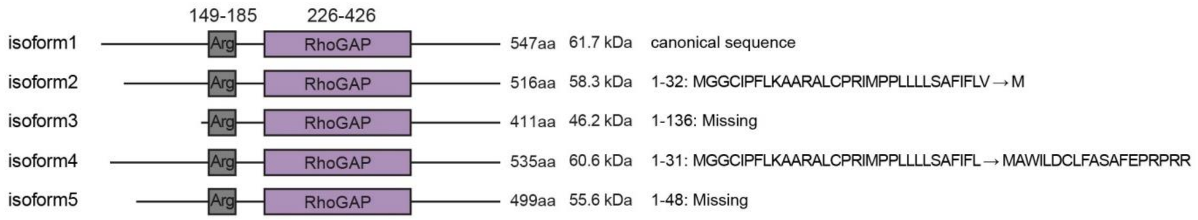

Supplementary Figure 1 Depiction of the five ARHGAP36 isoforms, based on UniProt entries (**Q6ZRI8-1 - Q6ZRI8-5)** as of March 2016.

Supplementary Figure 2

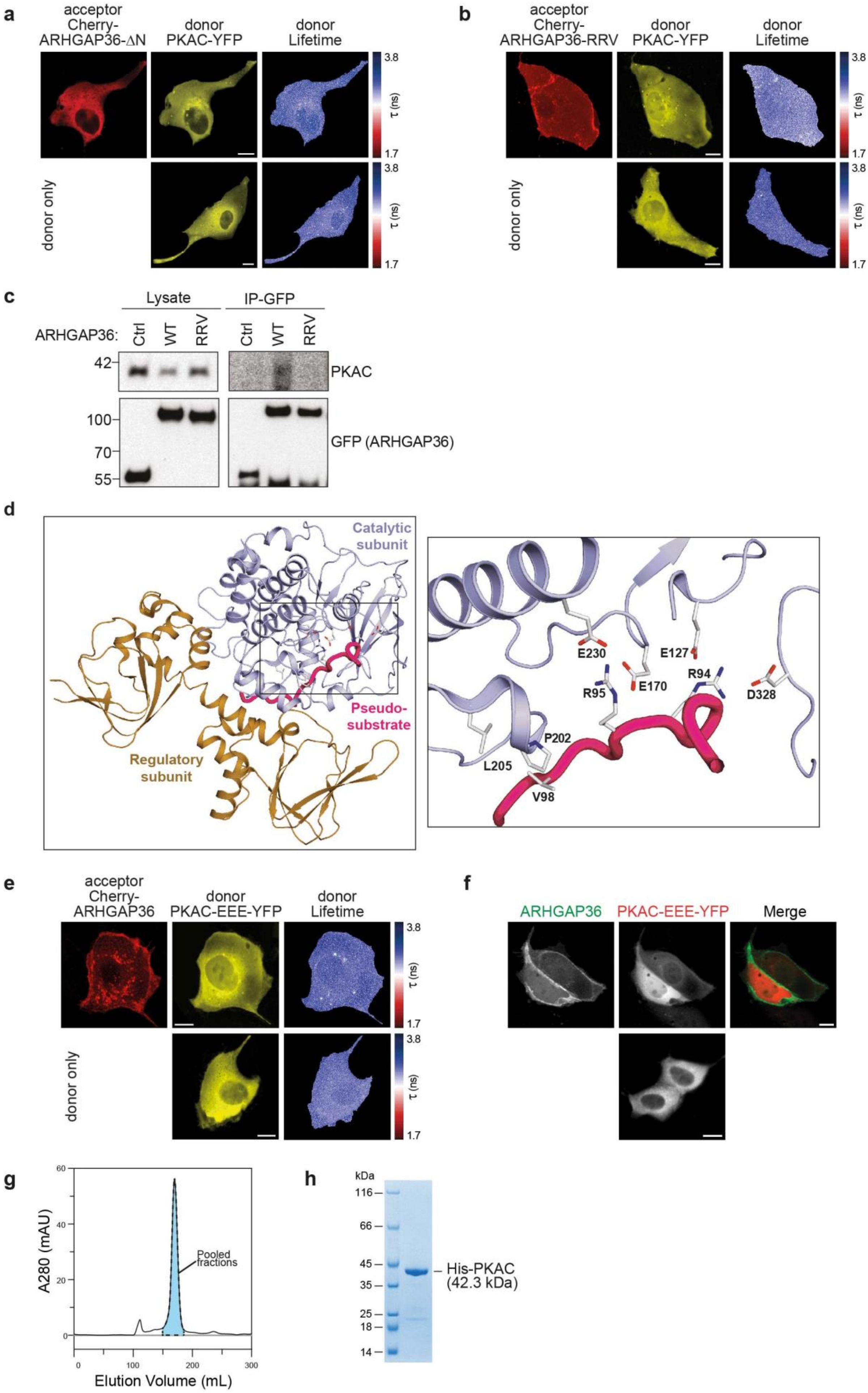

Supplementary Figure 2 (a) FLIM-FRET measurements in MDCK cells expressing PKAC-YFP (donor) either alone or together with mCherry-ARHGAP36-ΔN (acceptor), or **(b)** together with mCherry-ARHGAP36-RRV (acceptor). Shown are YFP and mCherry confocal images and the pseudocoloured donor fluorescence lifetime maps. Scale bars: 10 µm. **(c)** HEK293T cells were transfected with CFP-ARHGAP36, the RRV mutant or a CFP-Cherry control. Lysates were immunoprecipitated using a GFP antibody, and immunoblotted with GFP or PKAC antibodies. **(d)** Structure of the PKAC-PKARIIβ complex (in blue, sand, pdb 3TNP.pdb)[1]. The pseudosubstrate motif of the regulatory subunit is coloured in pink. The inset shows the three residues in the pseudosubstrate sequence (R94, R95, V98) that were mutated in the corresponding ARHGAP36 sequence, together with selected contacts in the catalytic subunit. **(e)** FLIM-FRET measurements in MDCK cells expressing PKAC-EEE-YFP (donor) either alone or together with mCherry-ARHGAP36 (acceptor). Shown are the YFP and mCherry confocal images and the pseudocoloured donor fluorescence lifetime maps. **(f)** Confocal live micrographs of MDCK cells expressing PKAC-YFP-EEE alone or together with CFP-ARHGAP36. Scale bars: 10 µm**. (g)** Size exclusion chromatogram (S75, 26/600) from the final purification step of His-PKAC. Fractions highlighted in blue were pooled and separated on an SDS-PAGE gel shown in **(h).**

Supplementary Figure 3

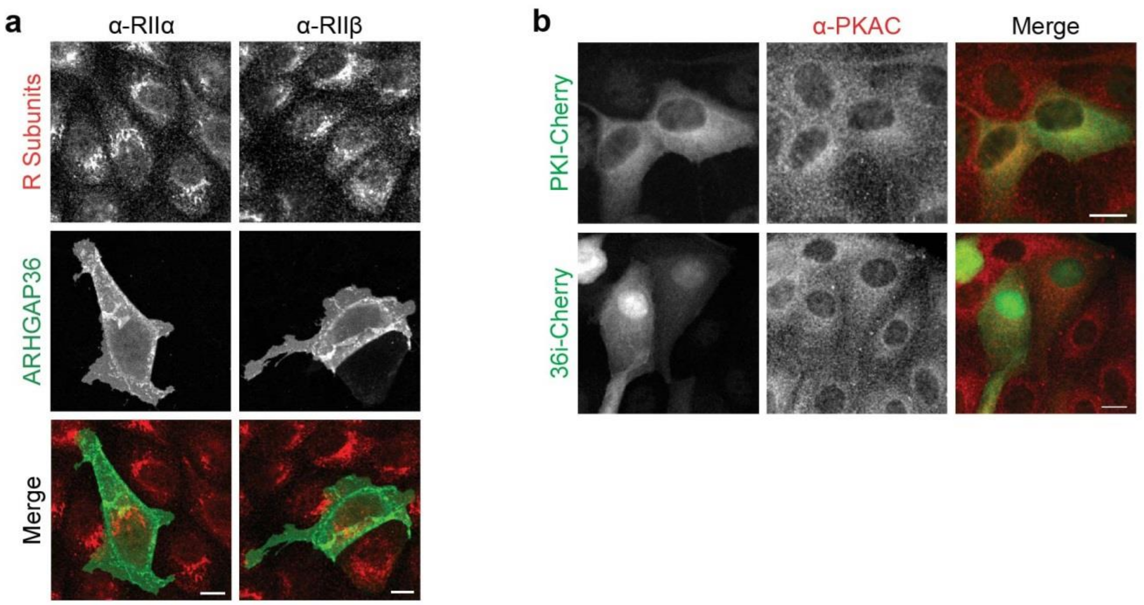

Supplementary Figure 3 (a) MDCK cells transfected with YFP-ARHGAP36 were fixed after 24 hours and subjected to immunofluorescence using antibodies against the indicated PKA regulatory (R) subunits. Scale bars: 10 μm. **(b)** Confocal micrographs of MDCK cells transfected with PKI-Cherry or 36i-Cherry, fixed and stained for endogenous PKAC. Scale bars: 10 μm. Both N- and C-terminally tagged 36i or PKI constructs thus do not affect endogenous PKAC protein levels.

Supplementary Figure 4

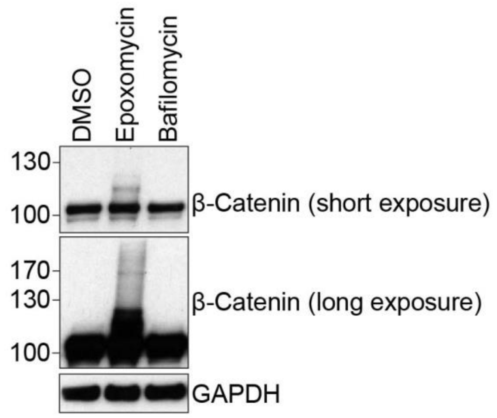

Supplementary Figure 4 HEK293T cells were treated for 6.5 hours with Epoxomicin (50 nM) or Bafilomycin (100 nM) before harvesting, the same time as in Figure 5a. Lysates were immunoblotted with the indicated antibodies. Epoxomicin causes the proteasomal substrate β-Catenin to accumulate dramatically over the treatment time.

Supplementary Figure 5

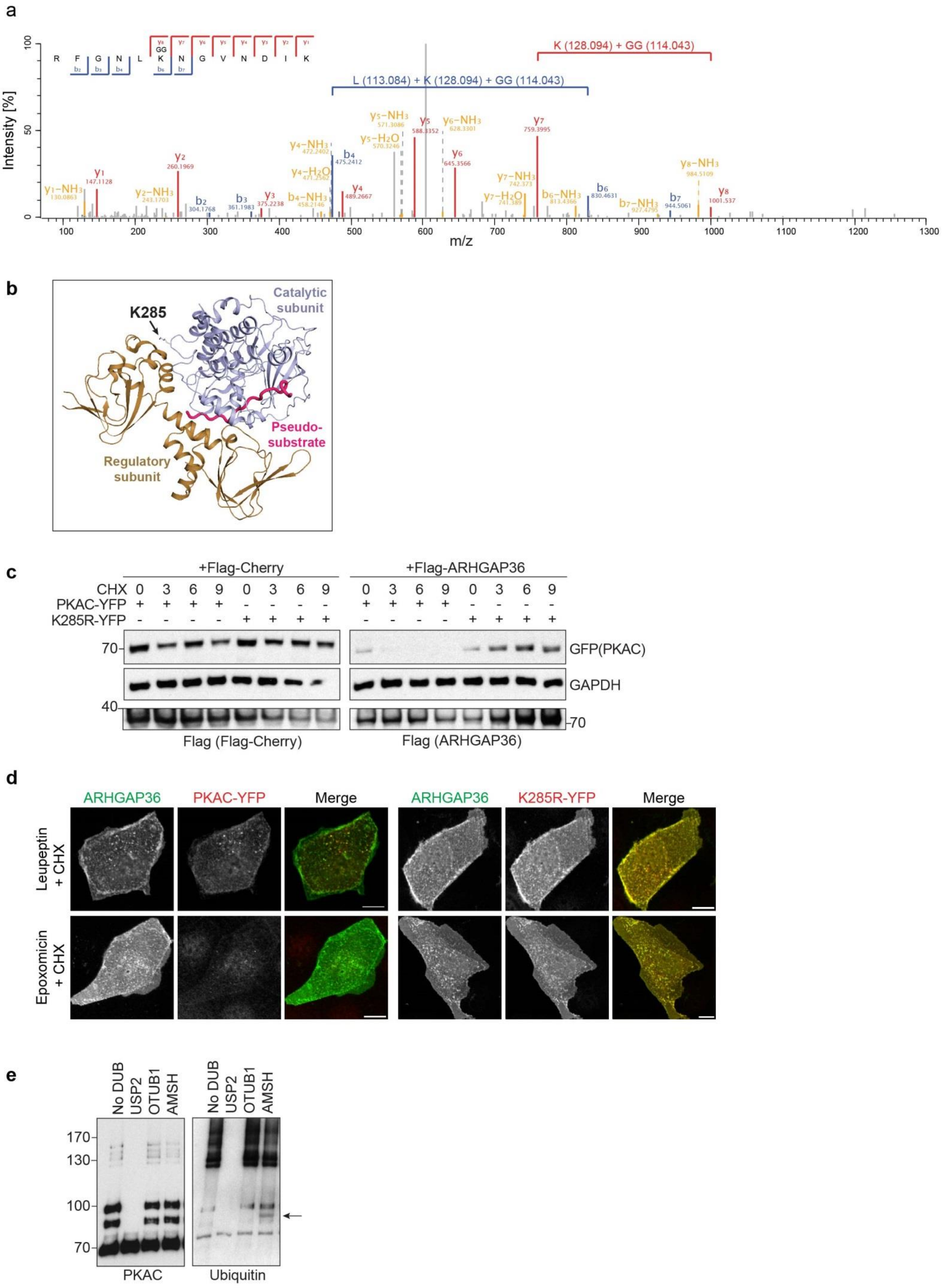

Supplementary Figure 5 (a) One out of two MS/MS spectra identifying the GlyGly modified peptide of PRKACA. Amino acid sequence, modified amino acid and the corresponding ions are shown. The mass shift introduced by the ubiquitination is annotated in both the b (blue) and y (red) series. **(b)** Structure of the PKAC-PKARIIβ complex, as in Figure S2d, showing the position of K285 on PKAC that undergoes ubiquitination. **(c)** HEK293T cells were transfected with PKAC-YFP or PKAC-K285R-YFP, and Flag-ARHGAP36 or Flag-Cherry control as indicated. 12 hours after transfection cells were treated with 50 µg/ml cycloheximide and harvested at the indicated time points. Lysates were immunoblotted with indicated antibodies. **(d)** MDCK cells were transfected with PKAC-YFP or PKAC-K285R-YFP and Flag-ARHGAP36 as in Fig. 6f. 12 hours after transfection, cells were pre-treated for 30 minutes with Leupeptin (500µM) or Epoxomicin (100nM) before cycloheximide (1µg/ml) addition for a further five hours. Cells were fixed and subjected to immunofluorescence using antibodies against GFP and Flag. Images were collected by confocal microscopy. Scale bars: 10 µm. **(e)** HEK293T cells were transfected with His-Ubiquitin, PKAC-YFP and Flag-ARHGAP36. Lysates was subjected to a single GFP IP, then divided and incubated for 45 minutes at 37°C with the indicated DUB. Eluates were immunoblotted with antibodies against PKAC or Ubiquitin. Arrow indicates cleaved ubiquitin. USP2, a non-selective DUB reliably removed the entire smear, confirming the modification as ubiquitin. While the K48-specific OTUB1 did not affect the ubiquitylation pattern, partial cleavage was seen with the K63-specific DUB AMSH.

Supplementary Figure 6

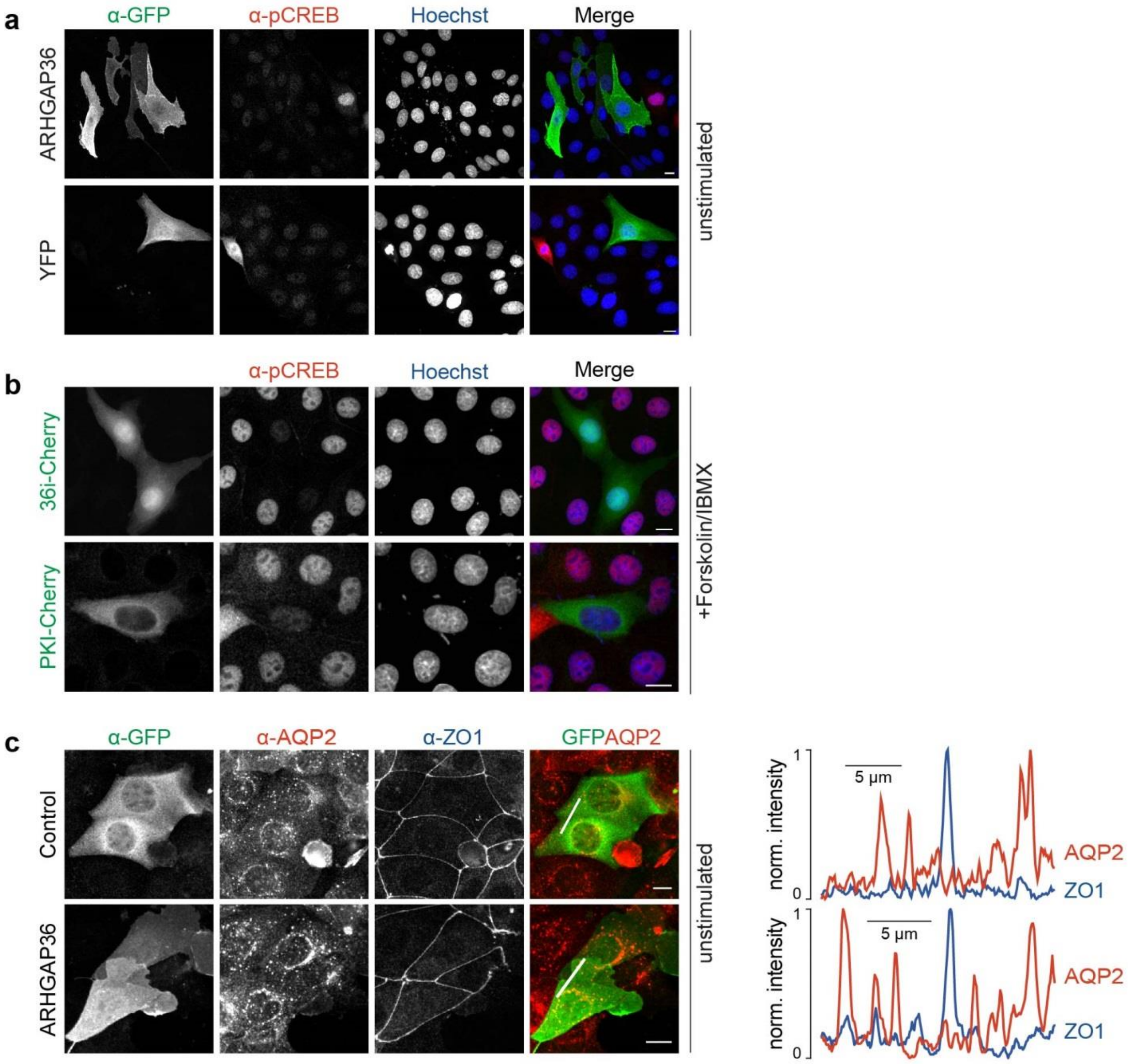

Supplementary Figure 6 (a) MDCK cells expressing YFP-ARHGAP36 or YFP control in low serum conditions were fixed without any stimulation and subjected to immunofluorescence using antibodies against GFP and phospho-CREB. Images were collected by confocal microscopy. Scale bars: 10 μm. **(b)** As in Figure 6b except cells were transfected with 36i-Cherry or PKI-Cherry. N- or C-terminal tagging therefore has no effect on the ability of 36i or PKI to inhibit PKA. **(c)** MCD4 cells stably expressing Aquaporin2 (AQP2) were transfected with YFP-ARHGAP36 or YFP control in low serum conditions. 24 hours post-transfection, without any stimulation, cells were fixed and subjected to immunofluorescence using antibodies against GFP, AQP2 and ZO-1. Images were collected by confocal microscopy. Scale bars: 10 μm. Line scan fluorescence intensity profiles are shown on the right. In red: AQP2, in blue: ZO-1.

Supplementary Figure 7

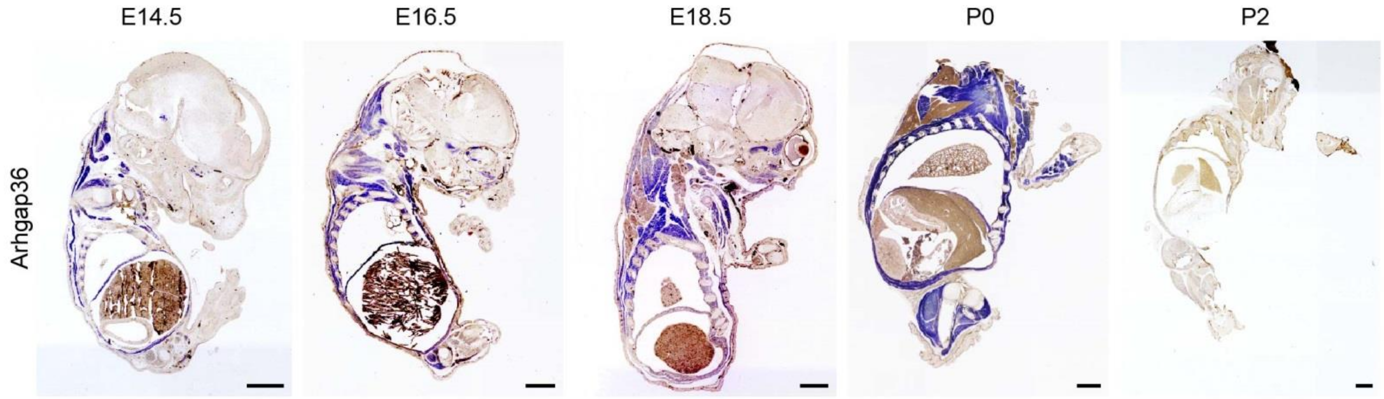

Supplementary Figure 7 *In situ* hybridization using an *Arhgap36*-specific probe on wild-type mice at the indicated developmental stages. Scale bars: 1 mm.

Supplementary Figure 8

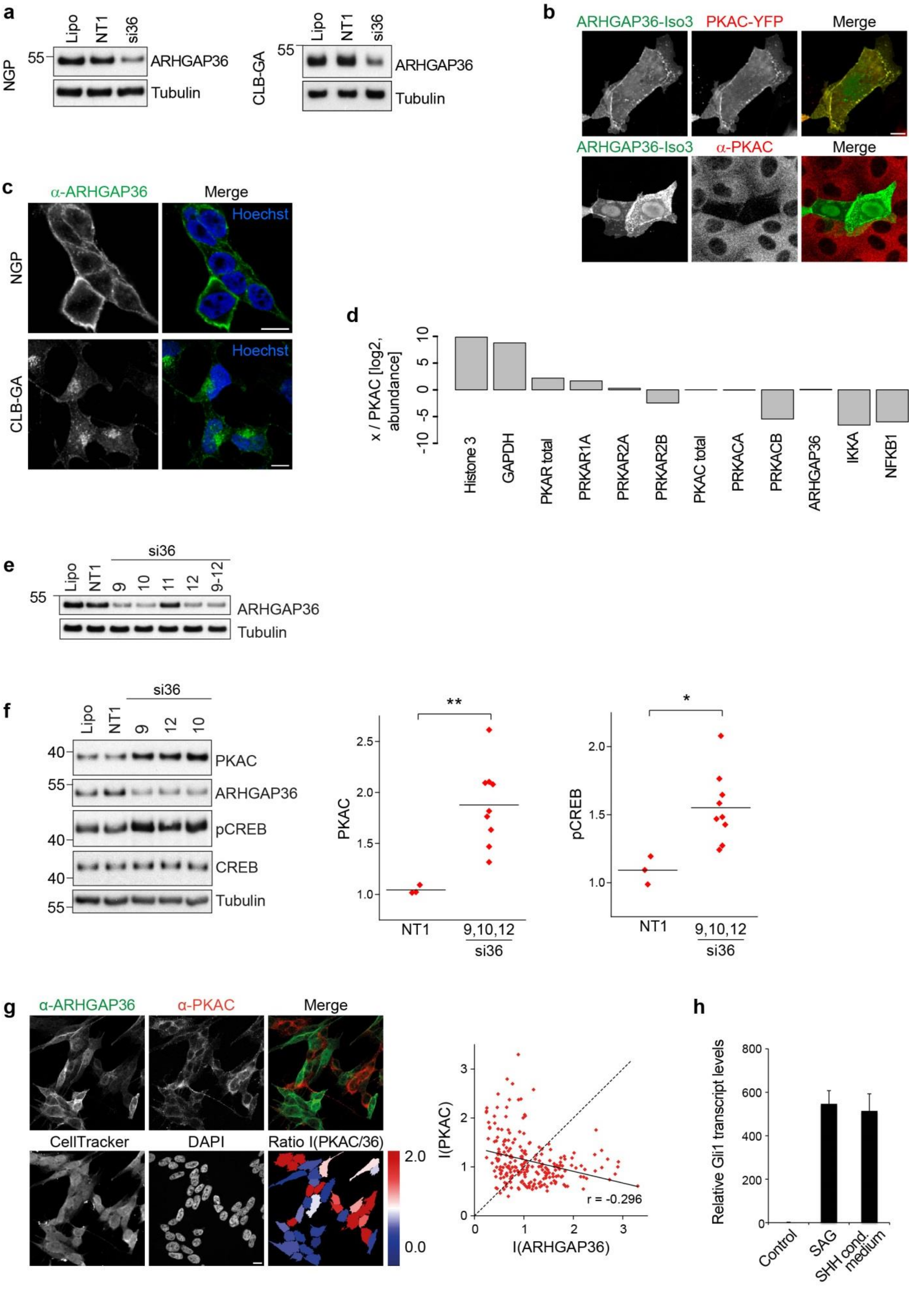

Supplementary Figure 8 (a) To confirm the specificity of the antibody, ARHGAP36 was knocked down using an siRNA SMARTpool (si36) in NGP or CLBGA cells for 24 or 48 hours respectively. Lipo: reagent only control, NT1: non-targeting oligo control. Lysates were immunoblotted with the indicated antibodies**. (b)** As isoform 3 seems to be predominantly expressed, we confirmed that it interacts with and degrades PKAC. Top panel: Confocal live cell micrographs of CFP-ARHGAP36-Iso3 overexpressed in MDCK cells together with PKAC-YFP, as in Fig. 1c. Bottom panel: MDCK cells transfected with YFP-ARHGAP36-Iso3 were fixed after 24 hours and subjected to immunofluorescence using antibodies against GFP and PKAC, as in Fig.4e. Images were collected by confocal microscopy. Scale bars: 10 μm. **(c)** Confocal micrographs of NGP or CLBGA cells subjected to immunofluorescence using an antibody against ARHGAP36. Scale bars: 10 μm. **(d)** IBAQ: Bar plot of log 2 abundance of the indicated proteins relative to PKAC total, the summed total of PRKACA and PRKACB. **(e)** The ARHGAP36 siRNA SMARTpool was deconvoluted into single oligos. NGP cells were treated with individual or pooled siRNA oligos (si36) against ARHGAP36 for 24 hours. Cells were stimulated with 10 µm Forskolin and 100 µm IBMX before harvesting. Lysates were immunoblotted with the indicated antibodies. Lipo: reagent only control. NT1: non-targeting oligo control. Oligo 11 was left out of subsequent experiments. **(f)** Representative immunoblot of NGP cells treated with individual siRNA oligos (si36) against ARHGAP36 for 24 hours. Cells were stimulated with 10 µm Forskolin and 100 µm IBMX before harvesting. Lysates were immunoblotted with the indicated antibodies. Lipo: reagent only control. NT1: non-targeting oligo control. PKAC and pCREB levels were densitometrically evaluated from three independent experiments and normalised to lipofectamine control. ** $p < 0.01$ or * $p < 0.05$ compared to NT1. **(g)** Confocal micrographs of CellTracker Deep Red treated NGP cells subjected to immunofluorescence using antibodies against ARHGAP36 and PKAC. Cells were segmented and the ratios of average PKAC over ARHGAP36 fluorescence intensities of single cells are shown in the pseudocolour images. Scale bar: 10 μm. The scatterplot shows the normalised single cell average intensity of PKAC plotted against ARHGAP36 (red dots, n=225 cells). Linear regression analysis (continuous line) indicates a negatively correlated distribution (Pearson's sample correlation coefficient r = -0.296). Dashed line indicates theoretical maximal positive correlation (r = 1). **(h)** qRT-PCR of Gli1 transcript levels in NIH3T3 cells treated with SAG (200 nM) or SHH-conditioned media (1:10) for 24 hours. Data shown as mean of three repeats +/- SEM.

Supplementary Figure 9

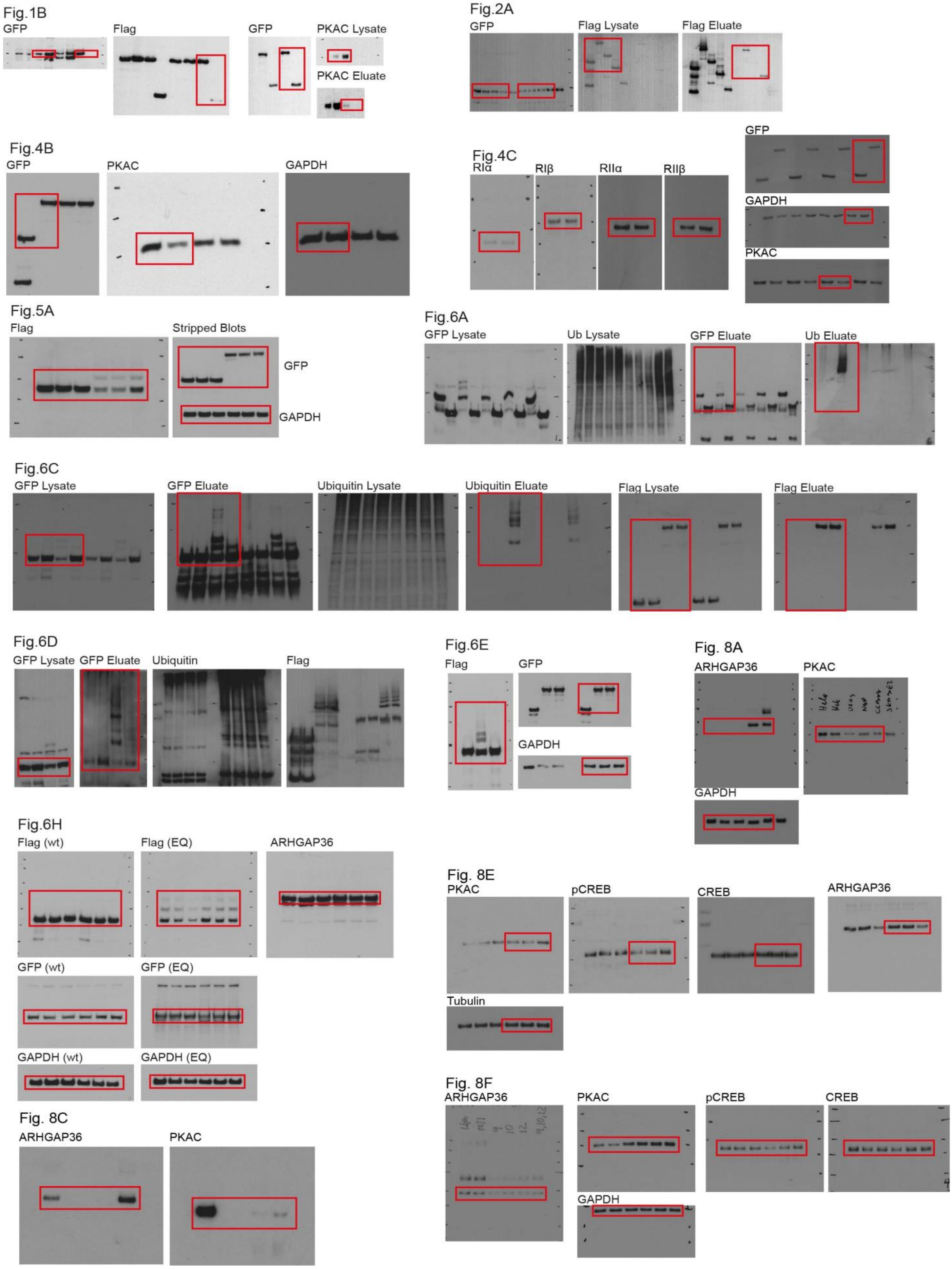

Supplementary Figure 9 Uncropped Western blots from all main figures.

Supplementary Figure 10

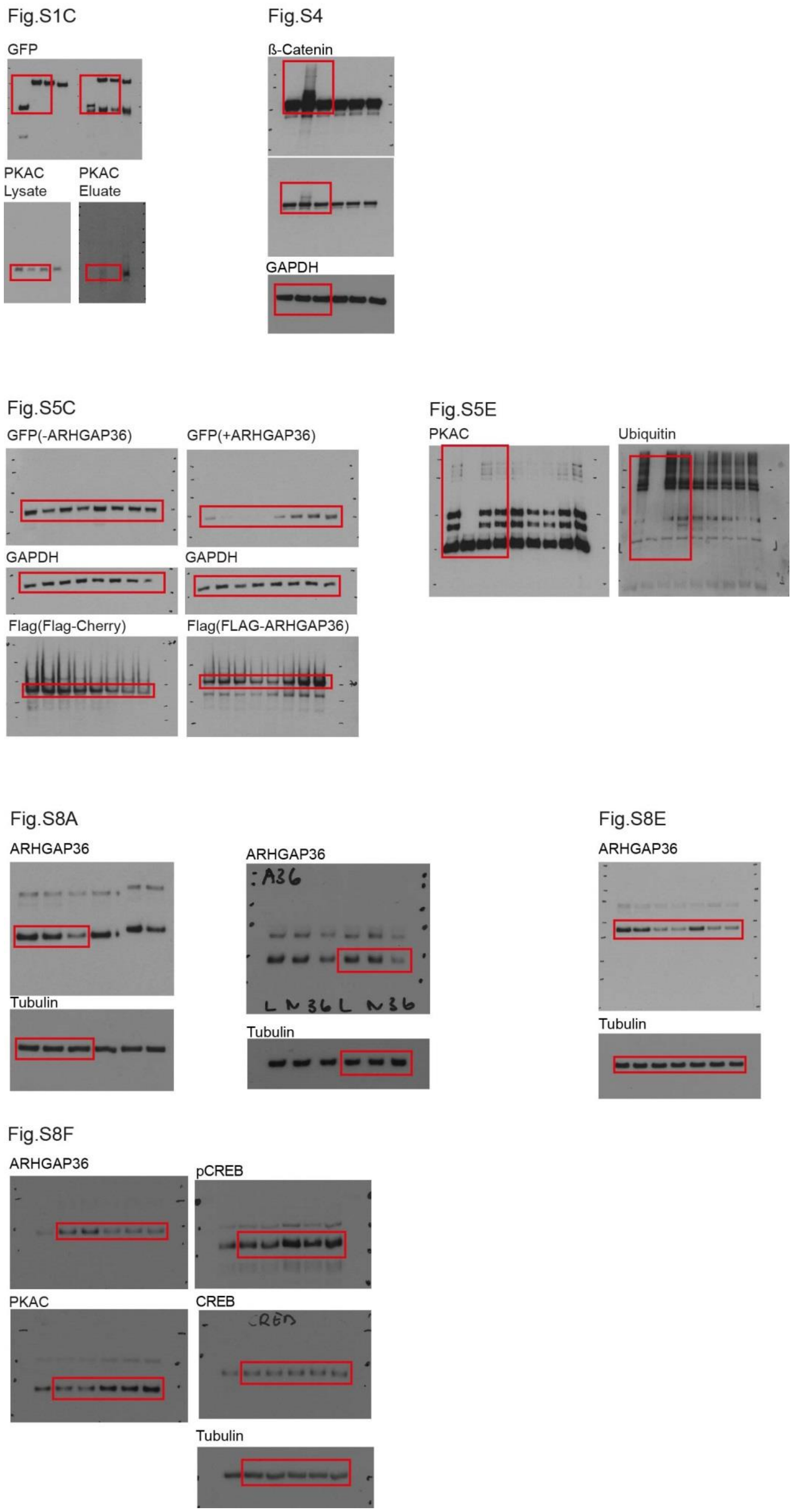

Supplementary Figure 10 Uncropped Western blots from all supplemental figures.

SUPPLEMENTARY REFERENCES

1. Zhang, P. *et al.* Structure and allostery of the PKA RIIbeta tetrameric holoenzyme. *Science* 335, 712-716 (2012).
